# Carvacrol-Loaded Chitosan Nanoparticles as a Multifunctional Nanotherapeutic Strategy Targeting Oxidative Stress, Inflammation, Apoptosis, and Genotoxicity in Nonalcoholic Fatty Liver Disease

**DOI:** 10.3390/antiox14121432

**Published:** 2025-11-28

**Authors:** M. Alfawaz, Ekramy M. Elmorsy, Ahmad Najem Alshammari, Noor A. Hakim, Najlaa M. M. Jawad, Soha A. Hassan, Manal S. Fawzy, Safya E. Esmaeel

**Affiliations:** 1 Department of Medical Laboratory Technology, College of Applied Medical Sciences, Northern Border University, Arar 91431, Saudi Arabia; mohammed.alfawaz@nbu.edu.sa (M.A.); ahmad.alshammari2@nbu.edu.sa (A.N.A.); 2 Center for Health Research, Northern Border University, Arar 73213, Saudi Arabia; ekramy.elmorsy@nbu.edu.sa; 3 Department of Forensic Medicine and Clinical Toxicology, Faculty of Medicine, Mansoura University, Mansoura 35516, Egypt; 4 Department of Clinical Nutrition, Faculty of Applied Medical Sciences, King Abdulaziz University, Jeddah 21589, Saudi Arabia; ohakim@kau.edu.sa (N.A.H.); nalsini@kau.edu.sa (N.M.M.J.); 5 Department of Biotechnology, Faculty of Applied Health Sciences Technology, October 6 University, 6th of October 12585, Egypt; soha.ahmed.dent@o6u.edu.eg; 6 Department of Physiology, Faculty of Medicine, Zagazig University, Zagazig 44519, Egypt; 7 Department of Physiology, College of Medicine, Northern Border University, Arar 91431, Saudi Arabia; safya.ebraheem@nbu.edu.sa

**Keywords:** high-fat diet, hepatic dysfunction, carvacrol, chitosan nanoparticles, antioxidants, anti-inflammatory effects

## Abstract

Nonalcoholic fatty liver disease (NAFLD) associated with high-fat diet (HFD) intake involves oxidative stress, inflammation, apoptosis, and genotoxicity. Carvacrol, a natural monoterpenoid phenol, exhibits potent antioxidant, anti-inflammatory, and cytoprotective properties, but its clinical application is limited by poor solubility and bioavailability. Chitosan nanoparticles, known for their biocompatibility and ability to enhance drug delivery, offer a promising nanotherapeutic platform for carvacrol delivery in NAFLD. Given the limited therapeutic options for NAFLD, there is a growing interest in nanotherapeutic strategies to enhance the delivery and efficacy of natural antioxidants. This study examined carvacrol-loaded chitosan nanoparticles (CRV-CNPs) in HFD-induced NAFLD. Sixty rats were assigned to six groups: control, CRV-treated (100 mg/kg), CRV-CNP-treated (100 mg/kg), HFD-fed, and two combination groups receiving HFD with either CRV or CRV-CNPs (100 mg/kg) for six weeks after 14 weeks on HFD. Liver function, metabolic markers, oxidative stress parameters, antioxidant enzyme levels, inflammatory and fibrotic mediators, apoptotic gene expression, genotoxicity indices, and histopathological changes were evaluated. CRV-CNPs showed greater efficacy than free carvacrol in ameliorating hepatic dysfunction and metabolic disturbances in HFD-fed rats. CRV-CNPs significantly reduced malondialdehyde, upregulated Nrf2, and elevated hepatic glutathione peroxidase, superoxide dismutase, catalase, and reduced glutathione. Inflammatory markers (NF-κB, iNOS, IL-1β, CRP) and transforming growth factor-beta were suppressed. Pro-apoptotic genes (*Bax*, *Caspase-3*) were downregulated, while antiapoptotic Bcl-2 was upregulated. CRV-CNPs also reduced DNA fragmentation and 8-hydroxy-2′-deoxyguanosine levels, indicating strong antigenotoxic effects. Histopathological and ultrastructural assessments revealed mitigated steatosis, preserved hepatic architecture, and maintained mitochondrial integrity. In conclusion, CRV-CNPs provide potent hepatoprotection by targeting oxidative stress, inflammation, apoptosis, and genotoxicity in NAFLD, demonstrating enhanced bioavailability, solubility, and sustained release, which support their potential as an advanced nanotherapeutic strategy for NAFLD management.

## 1. Introduction

**Figure 1:**
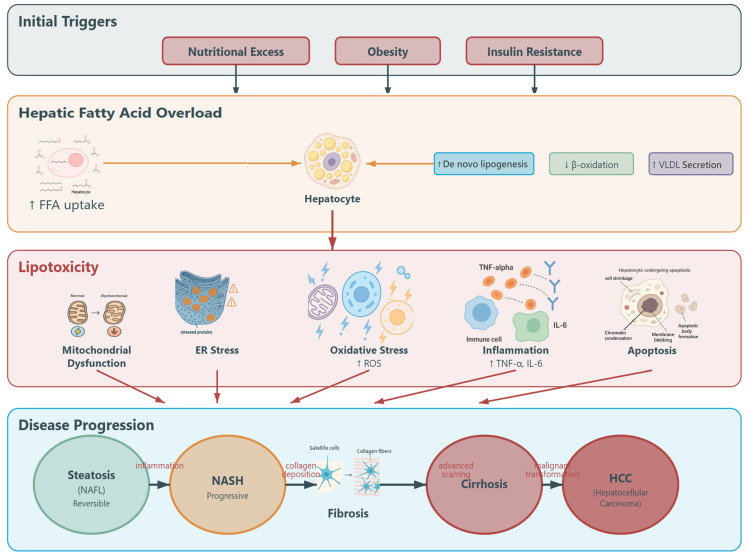
Schematic representation of the main biochemical mechanisms underlying nonalcoholic fatty liver disease (NAFLD) development and progression, highlighting key molecular pathways and targets of antioxidant/anti-inflammatory therapy, and biochemical pathways and progression of nonalcoholic steatohepatitis (NASH) to hepatocellular carcinoma (HCC). VLDL: Very low density lipoprotein, ER: Endoplasmic Reticulum.

Nonalcoholic fatty liver disease (NAFLD) is one of the most prevalent chronic liver disorders globally, affecting nearly 30% of the population [1]. Often regarded as the hepatic manifestation of metabolic syndrome, its severity correlates closely with obesity, hypertension, insulin resistance, and dyslipidemia [2]. Hepatic lipid accumulation in NAFLD acts not only as a marker of metabolic stress but also as a driver of pathological changes, ranging from simple steatosis to advanced cirrhosis [3]. NAFLD encompasses a spectrum of hepatic disorders, primarily classified as nonalcoholic fatty liver (NAFL) and nonalcoholic steatohepatitis (NASH). NAFL is characterized by a mild, reversible accumulation of lipids within hepatocytes, whereas NASH represents a more severe and progressive variant, often evolving into fibrosis, cirrhosis, and ultimately hepatocellular carcinoma [4,5] (Figure 1).

Lifestyle modification remains the first-line therapy for NAFLD management. Sustained weight loss of at least 5% can reduce hepatic steatosis and liver injury, while reductions over 7% are associated with histological improvement in NASH [6]. Additionally, regular physical activity enhances glucose and lipid metabolism, reduces steatosis, lowers liver enzymes, and supports the structural integrity of mitochondria and the liver [7]. Current pharmacological interventions for metabolic syndrome employ combinations of agents targeting associated comorbidities, yet no single therapy has consistently demonstrated efficacy for NAFLD treatment [8]. Accordingly, there is an urgent need to develop novel therapies targeting key pathogenic pathways, particularly those involving oxidative stress, apoptosis, and inflammatory signaling. In this context, natural products have attracted considerable attention for their antioxidant, antiapoptotic, and anti-inflammatory properties, which may help counter hepatic oxidative stress and inflammation in NAFLD [9].

Carvacrol (CRV; 5-Isopropyl-2-methylphenol) with a molecular formula: C_10_H_14_O, found in mountain thyme and oregano essential oils, has notable antioxidant, antiapoptotic, anti-inflammatory, and anticancer activities that often exceed those of synthetic antioxidants [10,11,12]. It is recognized as safe by the FDA and classified as a chemical flavoring by the European Commission [13]. Several experimental investigations have examined carvacrol’s hepatoprotective effects in NAFLD and related models. For example, Mohseni et al. showed that carvacrol administration ameliorated oxidative stress and inflammation in rats with diet-induced fatty liver, leading to significant reductions in serum ALT/AST and improved histological profiles [11]. Likewise, Cho et al. reported that carvacrol modulated lipid metabolism and reduced hepatic steatosis, partly by influencing gene expression involved in adipogenesis and inflammation [14]. Although these studies highlight carvacrol’s therapeutic promise in NAFLD, limitations in solubility, bioavailability, and sustained hepatic delivery remain significant barriers to clinical translation [15], and high doses may carry cytotoxic risks [16]. Therefore, nanoformulation strategies have thus been employed to enhance CRV’s stability, solubility, and bioavailability while minimizing toxicity [17].

Chitosan (CH), derived from chitin by deacetylation, is widely used as a nanomicro-delivery vehicle due to its biodegradability, biocompatibility, hydrophilicity, low toxicity, and straightforward nanoparticle fabrication [18,19]. Its versatility allows for production in various forms, including films, gels, membranes, fibers, and beads. It also promotes the transport of a wide range of molecules across mucosal barriers by temporarily modifying tight junctions [15]. CH is available in various molecular weights, allowing for precise control of nanoparticle or microparticle size [20]. Lower molecular weight CH is preferable for water solubility and nanoparticle production [21]. Moreover, the presence of free amino groups enables further functionalization for targeted delivery [15]. Importantly, nanoscale drug delivery systems offer several advantages over conventional formulations, including enhanced dissolution rates, increased solubility, and improved in vivo therapeutic outcomes, primarily due to their small particle size [22]. In this sense, the present study is distinguished by its novel integration of carvacrol with chitosan nanoparticles (CRV-CHNPs), designed to enhance solubility, stability, and bioefficacy. This nanotherapeutic approach specifically targets key biochemical mechanisms underlying HFD-induced NAFLD, including oxidative stress, inflammation, and apoptosis. By leveraging nanoparticle-mediated delivery, our strategy targets critical pathogenic pathways central to disease progression. Moreover, this study evaluates the hepatoprotective effects of CRV-CHNPs versus free carvacrol in a high-fat diet (HFD)-induced rat model, offering new insights into the therapeutic advantages and potential translational relevance of advanced nanodelivery systems in fatty liver disease.

Based on existing evidence that carvacrol exerts hepatoprotective effects and that chitosan nanoencapsulation improves bioavailability and cellular delivery, we hypothesized that CRV-CHNPs would provide superior protection against HFD-induced NAFLD in rats by more effectively ameliorating hepatic oxidative stress, inflammation, apoptosis, and genotoxicity compared to free carvacrol.

## 2. Materials and Methods

**Figure 2:**
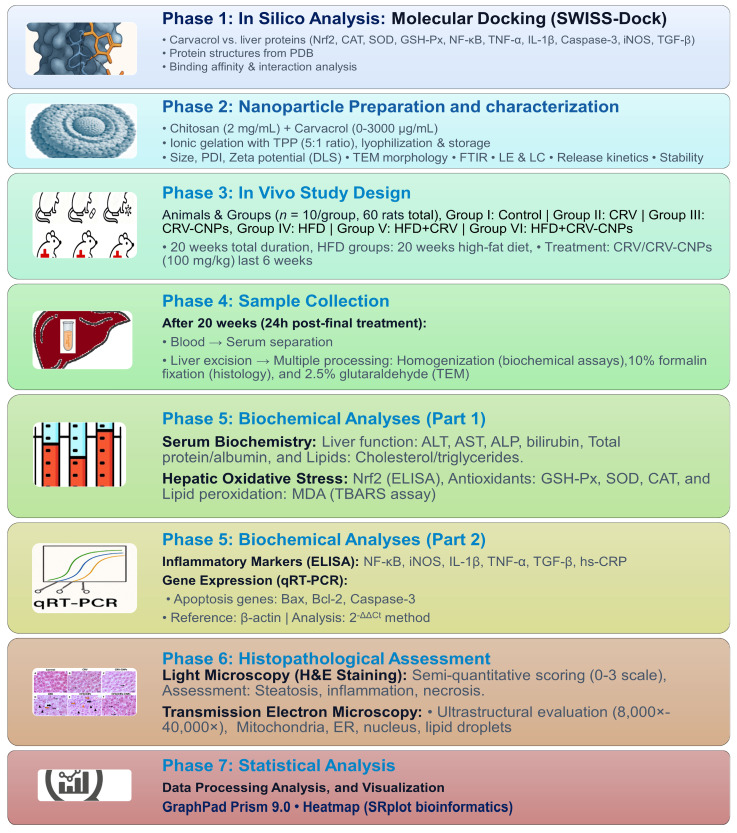
An overview of the experimental workflow in this study.

An overview of the experimental workflow, including molecular docking, nanoparticle preparation, in vivo interventions, and analytical endpoints, is summarized in Figure 2.

### 2.1. Molecular Docking

The SWISS-Dock server employed the EADock DSS engine and CHARMM force field to predict ligand–protein interactions involving Carvacrol and key liver molecules, including Nrf2, catalase (CAT), superoxide dismutase (SOD), glutathione peroxidase (GSH-Px), NF-κB, TNF-α, IL-1β, caspase-3, iNOS, and TGF-β. Crystal structures for all proteins were obtained from the Protein Data Bank. Subsequently, water molecules were removed, polar hydrogens were introduced, and standard energy minimization techniques were applied. Before docking, the 3D structure of carvacrol was optimized for energy efficiency. FullFitness and estimated binding free energy values were utilized to select the optimal binding poses. The SWISS-Dock and the Vienna model for analyzing and illustrating protein–ligand interactions, including hydrogen bonding and hydrophobic contacts, in high-resolution structural representations.

### 2.2. Preparation of Carvacrol (CRV)-Loaded Chitosan Nanoparticles (CRV-CHNPs)

**Figure 3:**
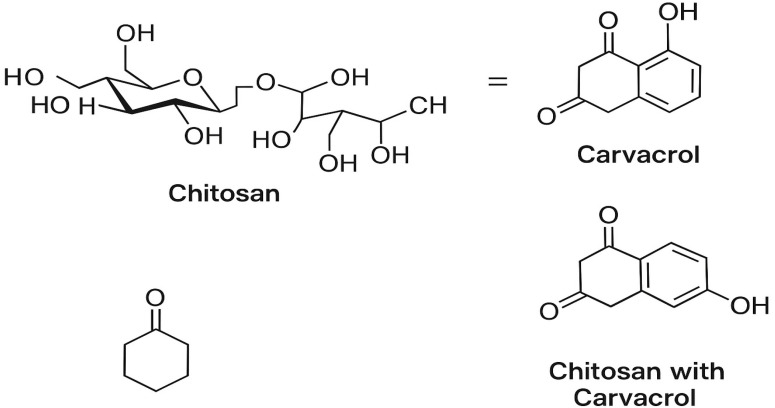
Chemical structures of chitosan and carvacrol, and schematic representation of their interaction within the carvacrol-loaded chitosan nanoparticle system.

Carvacrol (purity ≥ 99%) was purchased from AB Chem Company (Mansoura, Egypt). The chemical structures of chitosan and carvacrol, and their proposed interaction within the CRV-loaded chitosan nanoparticle system, are illustrated in Figure 3.

To prepare CRV-loaded chitosan nanoparticles (CRV-CHNPs), chitosan (2 mg/mL) was dissolved in 1% acetic acid, supplemented with 1% Tween 80, and sonicated for 15 min. The pH was adjusted to 5 with 2 NaOH, after which carvacrol (0–3000 µg/mL) was added, and the mixture was sonicated for 5 min. A solution of sodium tripolyphosphate in deionized water was gradually introduced to the chitosan solution while stirring continuously, maintaining a 5:1 (*w*/*w*) chitosan-to-crosslinker ratio. The suspension was magnetically stirred for one hour to enable cross-linking and stabilization. Nanoparticles were separated by centrifugation at 12,000× *g* for 20 min at 4 °C, rinsed twice with deionized water to remove free CRV and excess TPP, and then frozen at −80 °C. They were subsequently subjected to lyophilization.

Key characterization parameters, including zeta potential, hydrodynamic mean diameter, and PDI, were evaluated using a Zetasizer Nano ZS (Malvern Panalytical, Malvern, Worcestershire, UK). Physicochemical properties were assessed by measuring zeta potential in triplicate, and the Z-average was determined from diluted samples. The morphology of the nanoparticles was characterized by transmission electron microscopy (TEM; JEOL 2100, Tokyo, Japan) operated at 160 kV. According to the manufacturer (Sigma-Aldrich, St. Louis, MO, USA), the chitosan used had a molecular weight of 190–310 kDa and a deacetylation degree of 85%. These structural features play a crucial role in determining the encapsulation efficiency and stability of the formulations.

Encapsulation performance was assessed by calculating the loading efficiency (LE) and loading content (LC) of CRV in the nanoparticles. The calibration curve was obtained by analyzing a series of CRV standard solutions at 334 nm. Residual CRV in the supernatant was measured spectrophotometrically at 334 nm, and LE and LC were calculated using the equations below:

LE (%) = [CRV in nanoparticles / CRV added] × 100
LC (%) = [CRV in nanoparticles / Nanoparticle weight] × 100

CRV release in vitro was evaluated by the dialysis bag technique (MWCO 12–14 kDa) employing PBS (pH 7.4, 0.5% Tween-80) at 37 °C. At scheduled intervals of up to 48 h, samples were withdrawn, examined at 334 nm, and the cumulative release was calculated. Stability testing of lyophilized CRV-CHNPs was conducted at −20 °C, 4 °C, and 25 °C (dark) for 6 months, with evaluations of zeta potential, particle size, PDI, and encapsulation efficiency.

FTIR analysis was performed using an ATR-FTIR spectrometer (PerkinElmer, Waltham, MA, USA) in the range of 4000–400 cm^−1^ with a resolution of 4 cm^−1^ and 32 scans. Spectra were obtained for pure CRV, pure chitosan, a physical mixture of CRV and chitosan, and CRV-CHNPs.

### 2.3. Animals, Diets, and Experimental Protocol

Sixty adult male albino rats (average weight 179.73 ± 8.23 g) were obtained from the Animal Facility of the Faculty of Medicine, Mansoura University, Egypt. All experimental procedures adhered to the National Institutes of Health (NIH) Guide for the Care and Use of Laboratory Animals and the International Guiding Principles for Biomedical Research Involving Animals (1985). All animal procedures were revised and approved by the “Research Ethics Committee of the October 6 University, Egypt (approval code: 20251207)”.

Animals were acclimatized for two weeks before experimentation under standard laboratory conditions (temperature 21–25 °C, relative humidity 50–60%, and a 12 h light/dark cycle). All rats had unrestricted access to commercial rodent chow (Al Wadi Co., Giza, Egypt) and water throughout the study period. The standard rodent diet (Laboratory Rodent Diet 5001) contained 23% crude protein, 56% carbohydrates, 6% crude fiber, 8% ash, 4.5% crude fat, and 2.5% mineral mix, providing a caloric value of 3.36 kcal/g. The high-fat diet (HFD) was prepared by blending 35% beef tallow with 65% of the standard diet, yielding an energy density of 5.53 kcal/g. All diets were stored at 4 °C before use to preserve nutritional integrity. Rats were randomly allocated into six groups (*n* = 10 per group):

- Group I (Control): Received a standard laboratory rodent diet.
- Group II (CRV): Fed a standard diet and orally administered carvacrol (100 mg/kg body weight) for the final six weeks. The selected carvacrol dose was based on previous studies demonstrating its potent antioxidant and anti-inflammatory properties [23,24,25].
- Group III (CRV-CNPs): Received a standard diet and treated with carvacrol-loaded chitosan nanoparticles (100 mg/kg body weight) via oral gavage during the final six weeks.
- Group IV (HFD): Fed a high-fat diet (HFD) for 20 weeks according to the method described by Chang et al. [26].
- Group V (HFD + CRV): Fed an HFD for 20 weeks and co-administered carvacrol (100 mg/kg body weight) daily via oral gavage for the final six weeks.
- Group VI (HFD + CRV-CNPs): Fed an HFD for 20 weeks and co-administered CRV-loaded chitosan nanoparticles (100 mg/kg body weight) daily via oral gavage for the final six weeks.

The number of animals per group was determined according to current recommendations for preclinical in vivo studies using rodent models of metabolic and hepatic disorders [27]. Previous experimental reports and power analysis frameworks indicate that sample sizes of 8–12 rats per group are generally sufficient to detect biologically meaningful differences in biochemical and histopathological parameters with a statistical power of 0.8 and significance level of α = 0.05 [28]. This group size aligns with the ARRIVE 2.0 guidelines and internationally recognized ethical standards, ensuring appropriate statistical validity while adhering to the 3Rs principle (Reduction, Refinement, Replacement) of animal research [29]. The selected sample size also balanced ethical and practical considerations, allowing robust detection of treatment effects among six experimental groups while minimizing unnecessary animal use. All enrolled animals completed the study protocol with no exclusions, mortality, or data attrition. This design ensured reliable one-way ANOVA comparisons across groups and adhered to established preclinical standards for rigor, reproducibility, and research practice.

### 2.4. Tissue Sample Collection and Homogenization

Following an overnight fast, rats were euthanized by cervical dislocation 24 h after the final treatment. Blood samples were withdrawn from the hepatic portal vein into non-heparinized tubes, allowed to clot at room temperature, and centrifuged at 3000× *g* for 10 min to obtain serum. The resulting serum was aliquoted into Eppendorf tubes and immediately analyzed biochemically. Liver tissues were carefully excised, rinsed with ice-cold 1.15% potassium chloride (KCl) solution to remove residual blood, and stored at −20 °C until further biochemical analysis. For histopathological evaluation, representative liver sections were fixed in 10% neutral buffered formalin. For homogenate preparation, weighed portions of liver tissue were homogenized in 50 mmol/L Tris–HCl buffer (pH 7.4) using a Teflon-glass homogenizer at cold conditions. The homogenates were centrifuged at 10,000× *g* for 15 min at 4 °C, and the resulting supernatants were collected and stored at −80 °C for subsequent biochemical and enzymatic assays.

### 2.5. Body Weight and Liver Index

The body weight of each rat was recorded at the start of the study and again at the end of the 20 weeks. At sacrifice, the liver was rapidly excised, weighed, and used to calculate the liver index (liver weight/body weight × 100%) [30].

### 2.6. Serum Biochemistry and Liver Redox Assessment

Serum total protein, albumin, and globulin concentrations were determined colorimetrically according to the method described by Krohn [31]. Hepatic function markers, including alanine aminotransferase (ALT), aspartate aminotransferase (AST), and alkaline phosphatase (ALP), were assessed following the protocol of Goodla et al. [32]. At the same time, total bilirubin levels were quantified using the procedure described by Rosenthal et al. [33]. Serum lipid profile parameters, including total cholesterol and triglycerides, were determined following established colorimetric methods [34]. The catalog numbers of all chemicals and the accession IDs of the kits used in this study have been included as follows. Nuclear factor erythroid 2-related factor 2 (Nrf2) concentrations were measured using a rat-specific ELISA kit (Cat. No. E-EL-R1052; Elabscience^®^ Biotechnology Co., Ltd., Houston, TX, USA) in accordance with the manufacturer’s protocol. The levels of key hepatic antioxidant enzymes—glutathione peroxidase (GSH-Px), superoxide dismutase (SOD), and catalase (CAT)—were determined using colorimetric assay kits from BioDiagnostic^®^ (Giza, Egypt): GSH-Px (Cat. No. GSH-Px 2524), SOD (Cat. No. SOD 2521), and CAT (Cat. No. CA 2517).

Spectrophotometric readings were obtained using a BioTek^®^ microplate reader (Winooski, VT, USA) at 340 nm for GSH-Px (NADPH oxidation), 560 nm for SOD (nitroblue tetrazolium inhibition), and 510 nm for CAT (hydrogen peroxide decomposition). Lipid peroxidation was quantified by measuring malondialdehyde (MDA) levels using a thiobarbituric acid reactive substances (TBARS) assay kit (Cat. No. MD 2529; BioDiagnostic, Giza, Egypt) according to the manufacturer’s instructions.

### 2.7. Inflammatory Markers

Hepatic concentrations of key inflammatory mediators, including nuclear factor kappa B (NF-κB), inducible nitric oxide synthase (iNOS), interleukin-1β (IL-1β), and tumor necrosis factor-α (TNF-α), were quantified using rat-specific enzyme-linked immunosorbent assay (ELISA) kits according to the manufacturers’ protocols. The following kits were employed: NF-κB (Cat. No. E-EL-R0674) and iNOS (Cat. No. E-EL-R0520) from Elabscience^®^ Biotechnology Co., Ltd. (Houston, TX, USA), IL-1β (Cat. No. MBS702717) from MyBioSource (San Diego, CA, USA), and TNF-α (Cat. No. CEK1115) from Bio-Med Diagnostics (Milton Keynes, UK). Hepatic transforming growth factor-β (TGF-β) levels were determined using a commercial ELISA kit (Cat. No. E-UNEL-R0054, Elabscience^®^ Biotechnology Co., Ltd., Houston, TX, USA). In addition, high-sensitivity C-reactive protein (hs-CRP) concentrations were measured using a rat-specific CRP ELISA kit (eBioscience^®^, San Diego, CA, USA). All assays were performed in duplicate, and absorbance was measured at the appropriate wavelength using a microplate reader (BioTek Instruments, Winooski, VT, USA). The results were expressed as picograms or nanograms per milligram of protein in liver tissue homogenates.

### 2.8. RNA Isolation, cDNA Synthesis, and Quantitative Real-Time PCR

Liver tissue samples were homogenized in 1 mL of QIAzol Lysis Reagent (Qiagen, Hilden, Germany) using a TissueLyser II homogenizer (QIAGEN GmbH, Hilden, Germany). Chloroform was then added, and the mixture was centrifuged at 12,000× *g* for 15 min at 4 °C to achieve phase separation. The upper aqueous phase containing RNA was carefully collected into RNase-free microtubes. RNA was precipitated with isopropanol and recovered by centrifugation at 12,000× *g* for 10 min. The RNA pellet was subsequently washed with 75% ethanol, air-dried, and redissolved in nuclease-free water. Residual ethanol was removed by brief centrifugation at 7500× *g* for 5 min. The purity was evaluated based on the A260/A280 and A260/A230 absorbance ratios measured using a NanoDrop 2000 spectrophotometer (Thermo Fisher Scientific, Waltham, MA, USA). Values between 1.8 and 2.1 for A260/A280 and ≥1.8 for A260/A230 were considered acceptable, confirming minimal protein and solvent contamination. The integrity was verified by visualizing 28S and 18S rRNA bands on a 1% agarose gel. The concentration was calculated automatically from the absorbance at 260 nm, ensuring sufficient RNA yield for subsequent analysis.

Complementary DNA (cDNA) was synthesized from total RNA using the iScript™ cDNA Synthesis Kit (Bio-Rad, Hercules, CA, USA) following the manufacturer’s protocol. Quantitative real-time PCR (qRT-PCR) was performed using the iTaq™ Universal SYBR^®^ Green Supermix (Bio-Rad, Hercules, CA, USA, Cat. No. 172-5121) on a Rotor-Gene Q system (Qiagen, Hilden, Germany). Specific primers for apoptosis-related genes, Bax (Bcl-2-associated X), Bcl-2 (B-cell lymphoma-2), and Caspase-3 (cysteine–aspartic acid protease-3), were designed and synthesized by Macrogen (Seoul, South Korea) (Table 1). Primer stocks were reconstituted in RNase-free water and stored at −20 °C until use to preserve stability. The relative expression levels of the target genes were normalized to *β-actin* as the reference gene and calculated using the 2^−ΔΔCt^ method described by Livak and Schmittgen [35].

### 2.9. Histological Evaluation of Liver

Liver specimens designated for histological analysis were excised immediately after dissection and fixed in 10% neutral buffered formalin at a 20:1 fixative-to-tissue ratio (*v*/*v*) for 72 h. Fixed samples were dehydrated through graded ethanol solutions, cleared in xylene, and infiltrated with molten paraffin wax. Tissue blocks were embedded and sectioned using a rotary microtome at a thickness of 4–5 µm. Sections were mounted on glass slides, deparaffinized, rehydrated through a descending alcohol series, and stained with hematoxylin and eosin (H&E) for routine histological evaluation.

Histopathological examination was performed on three rats per group using a light microscope (Olympus BX53, Tokyo, Japan) at 400× magnification. For each animal, three representative slides were prepared, each containing three liver sections. Four non-overlapping fields were examined per section, yielding 12 microscopic fields per liver sample. The mean lesion score per rat was calculated by averaging the field scores for each animal. Semi-quantitative evaluation of hepatic steatosis followed the AASLD guidelines (Table 2). Lesions were scored semi-quantitatively from 0 (no lesion) to 3 (severe), and the mean score per rat was obtained by averaging all individual field scores. Tissue morphology was assessed by an experienced histopathologist blinded to the experimental groups to minimize bias. Semi-quantitative data were used to determine relative histological improvement across treatment groups.

### 2.10. Transmission Electron Microscopy (TEM)

For ultrastructural analysis, small liver specimens (~1 mm^3^) were immediately fixed in 2.5% glutaraldehyde prepared in 0.1 M phosphate buffer (pH 7.4) at 4 °C for 24 h to preserve cellular architecture. After primary fixation, tissues were rinsed three times with phosphate buffer and subsequently post-fixed in 1% osmium tetroxide (OsO_4_) for 2 h at 4 °C to enhance membrane contrast. Dehydration was performed using a graded ethanol series (50%, 70%, 90%, 95%, and 100%), followed by two rinses in acetone to ensure complete dehydration. Samples were infiltrated with a mixture of acetone and epoxy resin, embedded in pure epoxy resin (Epon 812 or equivalent), and polymerized at 60 °C for 48 h. Ultrathin sections (60–70 nm) were prepared using an ultramicrotome (Leica EM UC7, Vienna, Austria) equipped with a diamond knife. Sections were mounted on copper grids and counterstained sequentially with uranyl acetate (2%, 30 min) and lead citrate (10 min) to enhance structural contrast. The stained sections were examined using a JEOL JEM-2100 transmission electron microscope (JEOL Ltd., Tokyo, Japan) operated at an accelerating voltage of 160 kV. Digital micrographs were obtained at varying magnifications (8000×–40,000×) to evaluate hepatocellular ultrastructural changes, including mitochondrial morphology, endoplasmic reticulum integrity, nuclear condensation, and lipid droplet accumulation [36,37].

### 2.11. Statistical Analysis

All data were tested for normality using the Shapiro–Wilk test and for homogeneity of variances using Levene’s test before parametric analysis. Statistical comparisons among groups were performed using one-way analysis of variance (ANOVA) followed by Tukey’s post hoc multiple comparison test for pairwise group analysis (SAS software, version 9.4; SAS Institute Inc., Cary, NC, USA). Results are expressed as mean ± standard error of the mean (SEM), and differences were considered statistically significant at *p* < 0.05. Graphical representations were generated using GraphPad Prism 9.0 (GraphPad Software, San Diego, CA, USA). A hierarchical clustering heatmap was constructed to visualize comparative group relationships using the online bioinformatics platform SRplot—Science and Research Plotting Tool (https://www.bioinformatics.com.cn) (accessed on 12 October 2025) with the default visualization parameters.

## 3. Results

### 3.1. Molecular Docking Results

**Figure 4:**
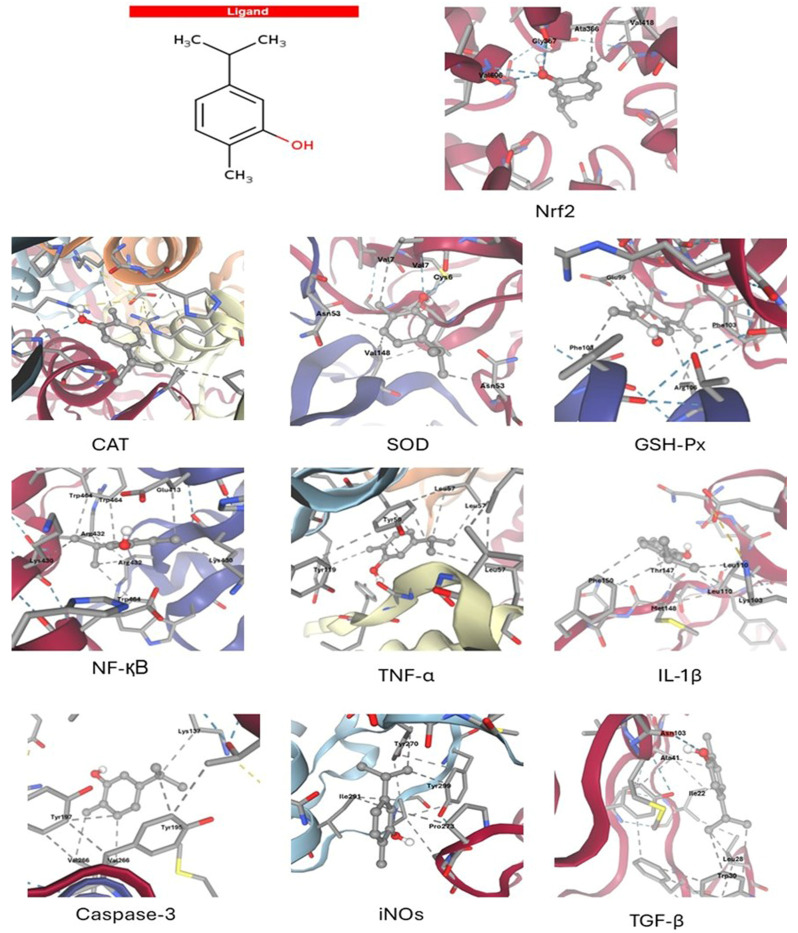
Molecular docking interactions of Carvacrol with key antioxidant, inflammatory, apoptotic, and fibrogenic targets involved in hepatic protection. The figure shows hydrogen bonds and hydrophobic interactions as dotted blue and gray lines, respectively. Also, major active-site residues contributing to ligand stabilization for each protein are shown. Nrf2: Nuclear factor erythroid 2–related factor 2; CAT: catalase; SOD: superoxide dismutase; GSH-Px: glutathione peroxidase; NF-κB: Nuclear Factor kappa-light-chain-enhancer of activated B cells; TNF-α: Tumor Necrosis Factor-alpha; IL-1β: Interleukin-1β; iNOs: inducible nitric oxide synthase; TGF-β: transforming growth factor-beta.

Molecular docking analysis revealed that Carvacrol exhibits a multifaceted interaction profile with antioxidant, inflammatory, and apoptotic signaling proteins relevant to hepatic protection. Carvacrol exhibited the highest binding affinity for GSH-Px (–6.0 kcal/mol) and Nrf2 (–5.7 kcal/mol) compared to other antioxidant targets. This was corroborated by several stabilizing interactions, including four hydrogen bonds with Nrf2 and significant hydrophobic contacts with critical residues such as Val606 and Gly367. Carvacrol exhibited moderate affinity for superoxide dismutase (SOD) (–5.4 kcal/mol) and catalase (CAT) (–5.0 kcal/mol), establishing limited hydrogen bonds while maintaining significant hydrophobic interactions with key catalytic residues, such as Val7 and His364. Carvacrol demonstrated a significant affinity for inflammatory mediators, particularly NF-κB (–6.3 kcal/mol), establishing eight hydrophobic interactions with key residues (Trp464, Arg432, Lys430). Docking with TNF-α (–5.6 kcal/mol) and IL-1β (–4.6 kcal/mol) supports the anti-inflammatory activity through interactions with critical aromatic and aliphatic residues. Carvacrol demonstrated moderate binding affinity for Caspase-3 (–4.1 kcal/mol) and TGF-β (–4.1 kcal/mol), primarily via hydrophobic interactions, suggesting a potential role in reducing apoptosis and profibrotic signaling (Figure 4 and Table 3).

### 3.2. Characterization of Carvacrol-Loaded Chitosan Nanoparticles (CRV-CNPs)

**Figure 5:**
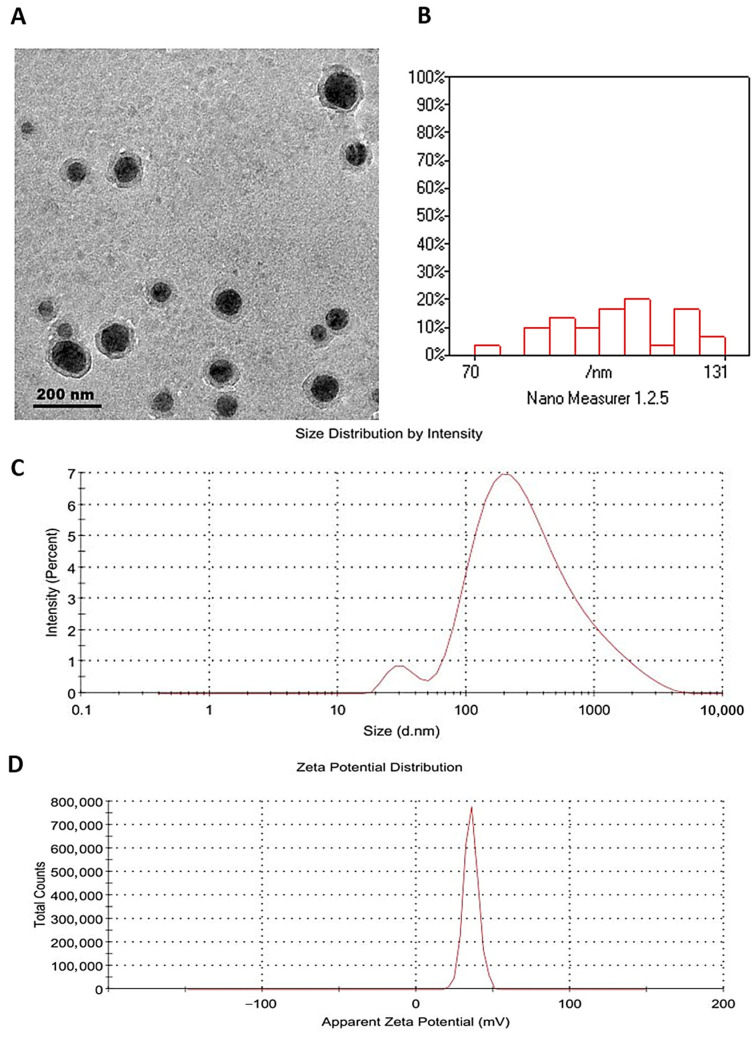
Characterization of Carvacrol-Loaded Chitosan Nanoparticles (CNPs): (**A**) TEM image showing nearly spherical particles; (**B**) particle size histogram indicating most particles range from 70 to 131 nm with good distribution; (**C**) size distribution by intensity; (**D**) zeta potential distribution. The nanoparticles exhibited a Z-average size of 192 nm, a PDI of 0.448, and a zeta potential of 35.9 mV.

Transmission electron microscopy revealed that the synthesized CRV-CNPs exhibited spherical morphology with uniform dispersion and smooth surfaces (Figure 5A). The particle size distribution determined from TEM micrographs ranged between 70 and 131 nm (Figure 5B), confirming nanoscale formation. In contrast, dynamic light scattering (DLS) analysis yielded a hydrodynamic diameter of 192 nm, a value higher than the particle diameter, which is attributed to the hydration layer and the Brownian motion of nanoparticles in the colloidal suspension. The polydispersity index (PDI) was measured at 0.448 (Figure 5C), suggesting acceptable monodispersity and uniform particle size distribution. The zeta potential of +35.9 mV (Figure 5D) indicated strong electrostatic repulsion among particles, supporting good colloidal stability of the nanoparticle suspension.

The entrapment efficiency of the formulation was 83.36%, verifying successful encapsulation of carvacrol within the chitosan matrix and consistency with earlier reports on chitosan–carvacrol nanocomposites. The formation of positively charged, spherical nanoparticles with a narrow size distribution supports their potential for enhanced bioavailability, controlled drug release, and improved therapeutic performance.

**Figure 6:**
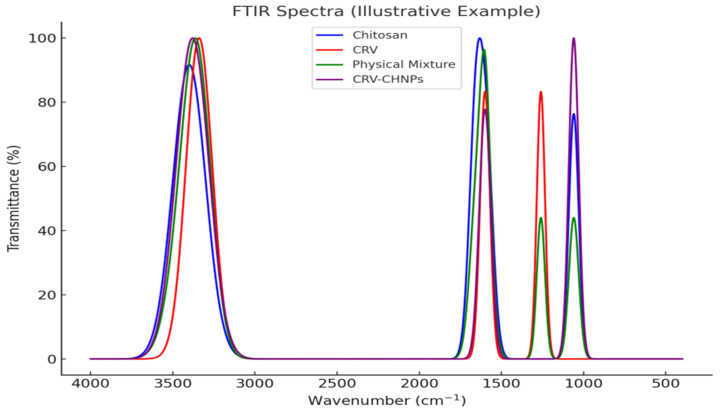
Attenuated Total Reflectance-Fourier Transform Infrared (ATR-FTIR) spectra of pure chitosan, carvacrol (CRV), their physical mixture, and CRV-CHNPs. Characteristic spectral shifts indicate the successful encapsulation of CRV.

ATR-FTIR spectra revealed distinct changes in functional group signals following nanoparticle formation. Specifically, a reduction in the intensity of the CRV phenolic band (~3340 cm^−1^) and a shift in the O–H/N–H stretching (~3400 cm^−1^) were observed in CRV-CHNPs, indicating hydrogen bonding interactions between carvacrol and chitosan, and confirming successful encapsulation (Figure 6).

### 3.3. Body Weight and Liver Index Results

**Figure 7:**
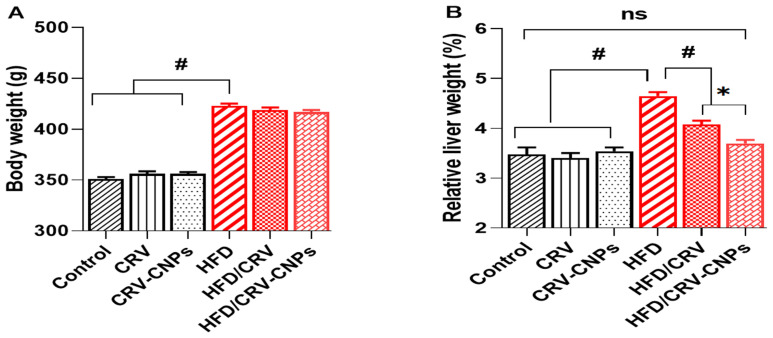
Effect of carvacrol (CRV) and CRV-loaded chitosan nanoparticles (CRV-CNPs) on body weight (**A**) and liver index (**B**) in rats receiving a high-fat diet. CRV: carvacrol (100 mg/kg body weight); CRV-CNPs: carvacrol-loaded chitosan nanoparticles (100 mg/kg body weight); HFD: high-fat diet; HFD/CRV: carvacrol (100 mg/kg body weight) + high-fat diet; HFD/CRV-CNPs: carvacrol-loaded chitosan nanoparticles (100 mg/kg body weight) + high-fat diet. Data are presented as mean ± standard deviation (SD) from *n* = 10 per group. Error bars depict both upper and lower limits of SD for each group. Significant group-wise differences are indicated by # (*p* < 0.05), * (*p* < 0.01), and ns (not significant).

As shown in Figure 7A, liver weight was significantly elevated in HFD-fed rats compared to animals receiving a normal diet or those treated with CRV or CRV-CNPs. Co-administration of CRV or CRV-CNPs slightly reduced liver weight; however, this reduction did not reach statistical significance compared to the HFD group.

In contrast, the liver index (ratio of liver weight to body weight) showed a more distinctive pattern. HFD-fed rats showed a significant increase in liver index compared with all control and treatment groups, consistent with hepatic lipid accumulation and steatosis commonly observed in HFD-induced NAFLD models. Co-treatment with CRV or CRV-CNPs markedly attenuated this increase, with CRV-CNPs exhibiting greater efficacy than free CRV in restoring the liver index to near-control values, with nonsignificant differences observed (Figure 7B).

### 3.4. Hepatic Function and Lipid Metabolism

The effects of carvacrol (CRV) and carvacrol-loaded chitosan nanoparticles (CRV-CNPs) on hepatic function and lipid metabolism indices in high-fat diet (HFD)-induced rats are presented in Table 4. HFD-fed rats demonstrated a significant decline in total serum protein levels, including both albumin and globulin fractions, compared with the normal control and groups receiving CRV or CRV-CNPs alone. Co-administration of CRV or CRV-CNPs significantly restored these parameters, with the most notable improvements observed in the HFD + CRV-CNPs group, where protein levels approached normal values. Conversely, no significant recovery was detected in the HFD + CRV group compared to the untreated HFD group.

Regarding hepatic enzyme activities, ALT, AST, ALP, and GGT were markedly elevated in HFD-fed rats relative to all control groups, reflecting hepatocellular injury. Both CRV and CRV-CNP supplementation significantly reduced these enzyme activities, with CRV-CNPs showing superior normalization, reaching levels that did not differ significantly from those of the normal control rats. Similarly, total bilirubin concentrations were elevated in the HFD group but significantly diminished following CRV-CNPs treatment, returning to baseline control levels.

### 3.5. Redox Status

**Figure 8:**
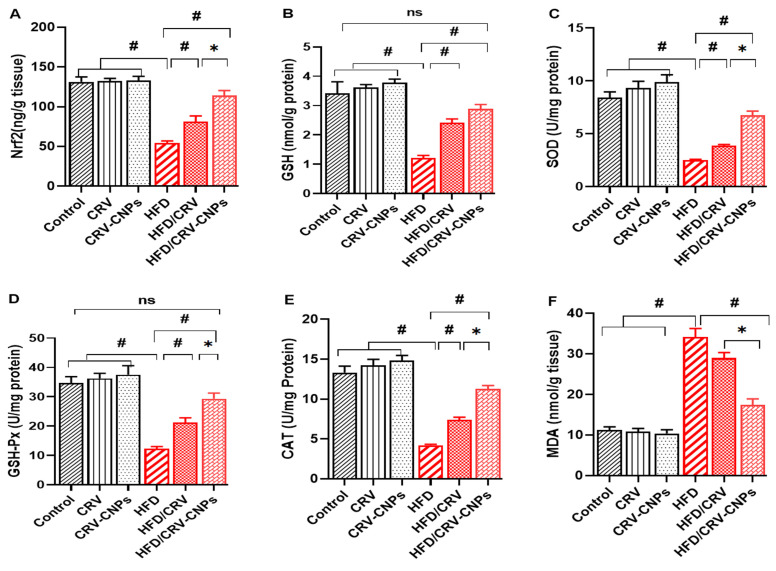
Effect of carvacrol (CRV) and CRV-loaded chitosan nanoparticles (CRV-CNPs) on hepatic redox status in rats receiving a high-fat diet (**A**–**F**). Nrf2: nuclear factor erythroid 2-related factor 2; GSH: glutathione; SOD: superoxide dismutase; GSH-Px: glutathione peroxidase; CAT: catalase; MDA: malondialdehyde. CRV: carvacrol (100 mg/kg body weight); CRV-CNPs: carvacrol-loaded chitosan nanoparticles (100 mg/kg body weight); HFD: high-fat diet; HFD/CRV: carvacrol (100 mg/kg body weight) + high-fat diet; HFD/CRV-CNPs: carvacrol-loaded chitosan nanoparticles (100 mg/kg body weight) + high-fat diet. # indicates significant difference compared to the HFD group; * indicates significant difference compared to the HFD/CRV-CNPs group; ns, nonsignificant.

As shown in Figure 7, Nrf2 activity (Figure 8A) was significantly reduced in HFD-fed rats compared with all control groups. Both CRV and CRV-CNP supplementation markedly restored Nrf2 levels, with the HFD + CRV-CNPs group exhibiting the greatest upregulation. This activation of Nrf2 was accompanied by parallel restoration of its downstream antioxidant system. Specifically, the levels of reduced glutathione (GSH) (Figure 8B) and the enzymatic activities of SOD, GSH-Px, and CAT (Figure 8C–E) were significantly decreased in the HFD group but notably increased after treatment with CRV or CRV-CNPs. Importantly, GSH and GSH-Px values in the nanoform-treated group were comparable to those of controls. Conversely, the HFD group exhibited a sharp rise in malondialdehyde (MDA), an indicator of lipid peroxidation (Figure 8F). This elevation was significantly reduced by CRV, with greater attenuation observed in CRV-CNP-treated rats.

### 3.6. Inflammation Response

**Figure 9:**
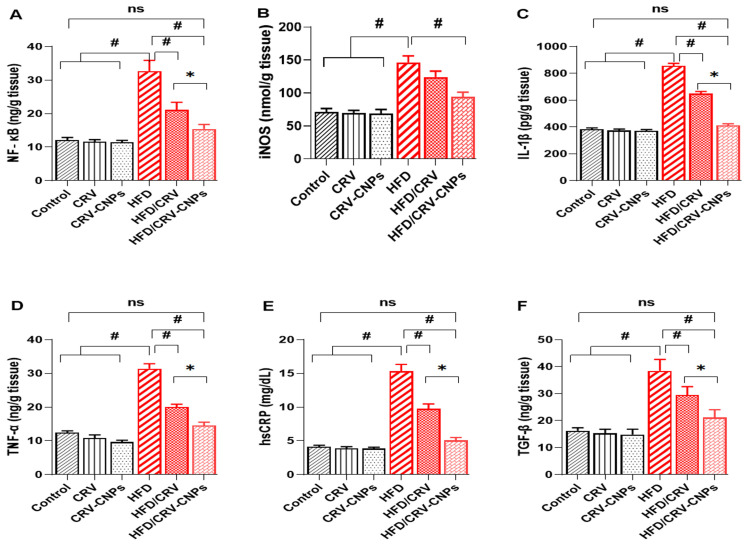
Effect of carvacrol (CRV) and CRV-loaded chitosan nanoparticles (CRV-CNPs) on hepatic inflammatory markers in rats receiving a high-fat diet (**A**–**F**). NF-kB, nuclear factor kappa B; iNOS, inducible nitric oxide synthase; IL-1β, interleukin-1β; TNF-α, tumor necrosis factor alpha; hs CRP, C-reactive protein; TGF-β, transforming growth factor-β. CRV: carvacrol (100 mg/kg body weight); CRV-CNPs: carvacrol-loaded chitosan nanoparticles (100 mg/kg body weight); HFD: high-fat diet; HFD/CRV: carvacrol (100 mg/kg body weight) + high-fat diet; HFD/CRV-CNPs: carvacrol-loaded chitosan nanoparticles (100 mg/kg body weight) + high-fat diet. # indicates significant difference compared to the HFD group; * indicates significant difference compared to the HFD/CRV-CNPs group; ns, nonsignificant.

As illustrated in Figure 9, hepatic inflammatory biomarkers were markedly elevated in HFD-fed rats. Levels of NF-κB (Figure 9A), iNOS (Figure 9B), IL-1β (Figure 9C), and TNF-α (Figure 9D) increased significantly compared with the normal control and groups receiving CRV or CRV-CNPs alone, indicating activation of hepatic inflammation caused by lipid overload. Treatment with CRV or CRV-CNPs significantly mitigated this response, with CRV-CNPs exhibiting superior efficacy. Notably, NF-κB, IL-1β, and TNF-α levels in the HFD + CRV-CNPs group were comparable to those in the normal control, demonstrating the nanoformulation’s enhanced ability to suppress the NF-κB/iNOS/IL-1β axis more efficiently than free CRV. Regarding systemic inflammation, C-reactive protein levels (Figure 9E) were significantly increased in HFD-fed rats, consistent with aggravated hepatic inflammation. Both CRV and CRV-CNPs significantly reduced CRP levels, with the strongest reduction in the HFD + CRV-CNPs group, restoring values near those of the normal control. Similarly, transforming growth factor-β (TGF-β) (Figure 9F) was significantly elevated in HFD-fed rats, reflecting early fibrotic activation. Co-treatment with CRV or CRV-CNPs significantly reduced TGF-β levels, with the CRV-CNPs formulation producing the greatest decline, approaching control levels.

### 3.7. Apoptosis-Related Genes

**Figure 10:**
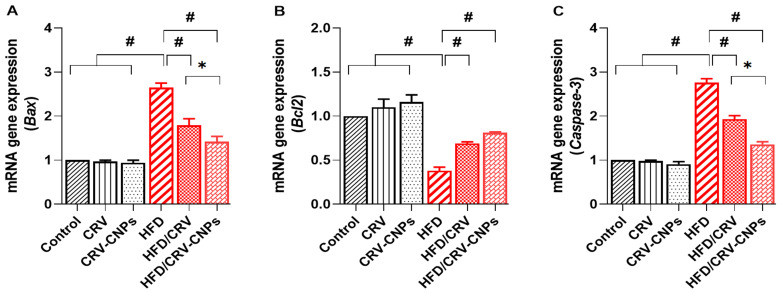
Effect of carvacrol (CRV) and CRV-loaded chitosan nanoparticles (CRV-CNPs) on Hepatic apoptotic gene expression in rats receiving a high-fat diet (**A**–**C**). *Bax*: Bcl-2-associated X protein; *Bcl2*: B-cell lymphoma 2; *Caspase 3*: cysteine–aspartic acid protease-3. CRV: carvacrol (100 mg/kg body weight); CRV-CNPs: carvacrol-loaded chitosan nanoparticles (100 mg/kg body weight); HFD: high-fat diet; HFD/CRV: carvacrol (100 mg/kg body weight) + high-fat diet; HFD/CRV-CNPs: carvacrol-loaded chitosan nanoparticles (100 mg/kg body weight) + high-fat diet. # indicates significant difference compared to the HFD group; * indicates significant difference compared to the HFD/CRV-CNPs group.

As shown in Figure 10, the expression of hepatic apoptosis-regulating genes was markedly altered in HFD-fed rats compared with the control groups. Specifically, *Bax* (Figure 10A) and *Caspase-3* (Figure 10C) mRNA levels were significantly upregulated, while *Bcl-2* expression (Figure 10B) was significantly downregulated, indicating enhanced pro-apoptotic signaling and compromised hepatocellular protection.

Treatment with CRV or CRV-CNPs substantially reversed the HFD-induced apoptotic imbalance, as evidenced by upregulation of *Bcl-2* and significant downregulation of *Bax* and *Caspase-3* expression relative to the HFD group. Notably, the CRV-CNP-treated rats exhibited a more pronounced modulation of apoptotic gene expression than those receiving free CRV, highlighting the nanoparticle formulation’s superior antiapoptotic efficacy. Furthermore, rats administered CRV or CRV-CNPs alone showed no significant difference from the control group, confirming the safety and non-cytotoxic nature of the treatment.

### 3.8. Hepatic DNA Damage Biomarkers

**Figure 11:**
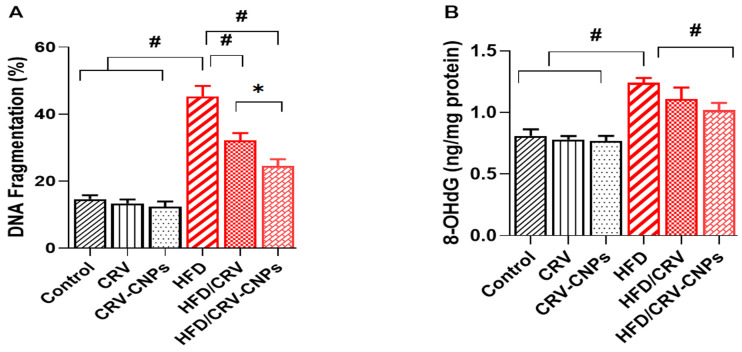
Effect of carvacrol (CRV) and CRV-loaded chitosan nanoparticles (CRV-CNPs) on Hepatic DNA fragmentation and 8-hydroxy-2′-deoxyguanosine (8-OHdG) levels in rats receiving a high-fat diet (**A**,**B**). CRV: carvacrol (100 mg/kg body weight); CRV-CNPs: carvacrol-loaded chitosan nanoparticles (100 mg/kg body weight); HFD: high-fat diet; HFD/CRV: carvacrol (100 mg/kg body weight) + high-fat diet; HFD/CRV-CNPs: carvacrol-loaded chitosan nanoparticles (100 mg/kg body weight) + high-fat diet. # indicates significant difference compared to the HFD group; * indicates significant difference compared to the HFD/CRV-CNPs group.

As shown in Figure 11, HFD-fed rats exhibited a significant elevation in hepatic DNA fragmentation, demonstrating increased genomic instability caused by lipid overload. Co-treatment with CRV or CRV-CNPs significantly reduced DNA fragmentation, with CRV-CNPs showing the most pronounced protective effect (Figure 11A). Similarly, hepatic concentrations of 8-hydroxy-2′-deoxyguanosine (8-OHdG), a well-established biomarker of oxidative DNA damage, were markedly increased in HFD-fed rats compared with controls. Treatment with CRV or CRV-CNPs significantly decreased 8-OHdG levels, with the lowest values observed in the HFD + CRV-CNPs group (Figure 11B).

### 3.9. Histopathological Examination

**Figure 12:**
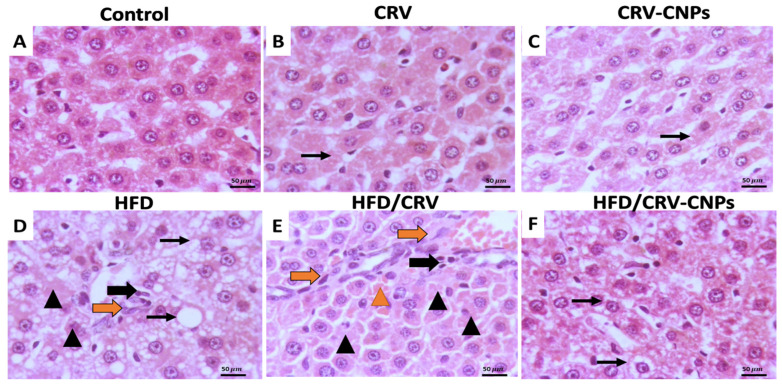
Representative photomicrograph of hepatic parenchyma of different treatment groups. (**A**) Control group; (**B**) CRV: carvacrol (100 mg/kg body weight); (**C**) CRV-CNPs: carvacrol-loaded chitosan nanoparticles (100 mg/kg body weight); (**D**) HFD: high-fat diet; (**E**) HFD/CRV: carvacrol (100 mg/kg body weight) + high-fat diet; and (**F**) HFD/ CRV-CNPs: carvacrol-loaded chitosan nanoparticles (100 mg/kg body weight) + high-fat diet. Thin arrows indicate hepatocellular swelling with vacuolation; black thick arrows indicate inflammation; orange thick arrow indicates fibrosis; arrowheads indicate hepatocellular necrosis; orange arrowhead indicates minor hemorrhage. magnification 400×; scale bar = 50 μm.

Microscopic evaluation revealed that administration of CRV or CRV-CNPs did not induce any histopathological alterations in hepatic tissues. The lobular architecture remained intact, the central veins appeared normal, and hepatocytes were polygonal with centrally located nuclei and eosinophilic cytoplasm (Figure 12A–C). In contrast, HFD-fed rats exhibited severe diffuse steatosis, characterized by numerous intracellular lipid vacuoles of varying sizes that displaced nuclei toward the cell periphery. Livers also showed scattered necrotic hepatocytes, diminished cellular detail, and mild perivascular inflammatory infiltration (Figure 12D). Liver sections from HFD + CRV-treated rats displayed mild-to-moderate hepatocellular dissociation, scattered necrotic cells, and focal to bridging portal fibrosis, accompanied by low inflammatory infiltrates and occasional hemorrhagic foci separating hepatic cords (Figure 12E). Conversely, the HFD + CRV-CNPs group exhibited only mild swelling and vacuolization of hepatocytes, with largely preserved hepatic cords and lobular organization (Figure 12F).

**Figure 13:**
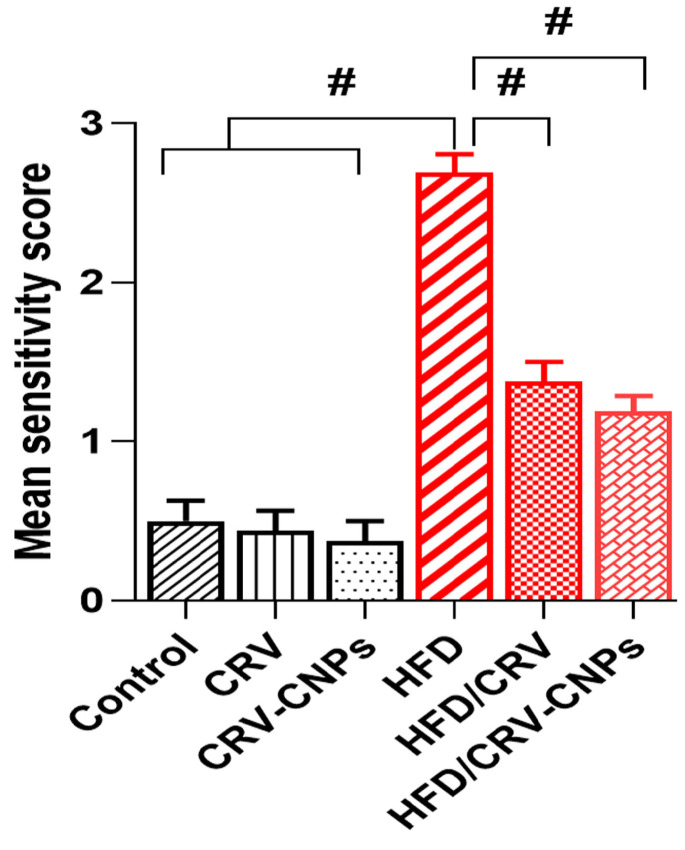
Histopathological severity scores of hepatic injury in control and experimental groups. CRV: carvacrol (100 mg/kg body weight); CRV-CNPs: carvacrol-loaded chitosan nanoparticles (100 mg/kg body weight); HFD: high-fat diet; HFD/CRV: carvacrol (100 mg/kg body weight) + high-fat diet; HFD/CRV-CNPs: carvacrol-loaded chitosan nanoparticles (100 mg/kg body weight) + high-fat diet. # indicates a significant difference compared to the HFD group.

Quantitatively, histopathological severity scores were significantly higher in the HFD group compared with controls. In contrast, CRV or CRV-CNPs administration markedly reduced these scores, with the most substantial improvement observed in the CRV-CNPs-treated rats (Figure 13).

### 3.10. Ultrastructural Examination

**Figure 14:**
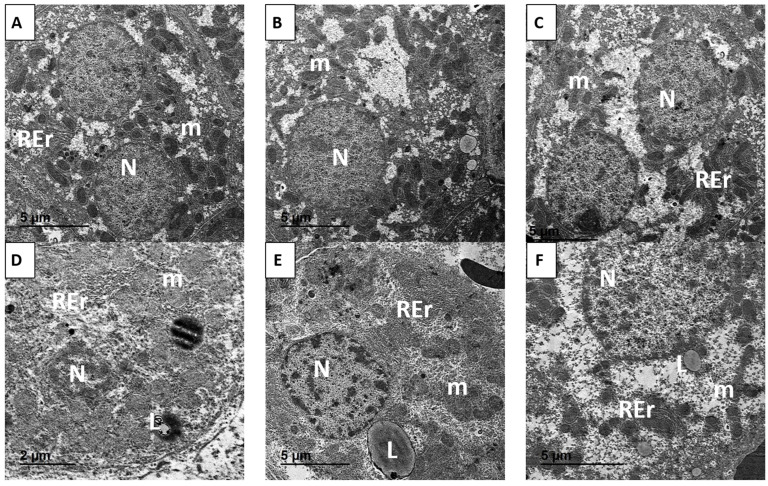
Representative photomicrograph of liver ultrastructure from control and different experimental groups. (**A**) control group; (**B**) CRV group; (**C**) CRV-CNPS; (**D**) HFD; (**E**) HFD/CRV; (**F**) HF/CRV-CPNPs (N) nucleus, (m) mitochondria, (REr) rough endoplasmic reticulum, (L) variable-sized lipid droplet.

As shown in Figure 14A–C, hepatocytes from the control, CRV-, and CRV-CNP-treated rats retained normal ultrastructural organization. Cells displayed well-preserved rough endoplasmic reticulum (rER), intact mitochondrial morphology, and spherical nuclei with distinct nucleoli, indicating maintained cellular homeostasis. In contrast, hepatocytes from HFD-fed rats showed severe ultrastructural alterations (Figure 14D). These included swollen mitochondria with disrupted cristae, dilated rER cisternae, cytoplasmic disintegration, chromatin condensation, irregular nuclear contours, and extensive cytoplasmic vacuolation, hallmarks of lipid-induced organelle stress and hepatocellular degeneration. Co-treatment with CRV (Figure 14E) or CRV-CNPs (Figure 14F) markedly alleviated these degenerative changes. Hepatocytes in the CRV-CNPs group exhibited largely preserved mitochondria and rER, intact nuclear membranes, and only minimal cytoplasmic vacuolation, demonstrating superior ultrastructural preservation compared with free CRV treatment.

### 3.11. Multivariable Analysis

**Figure 15:**
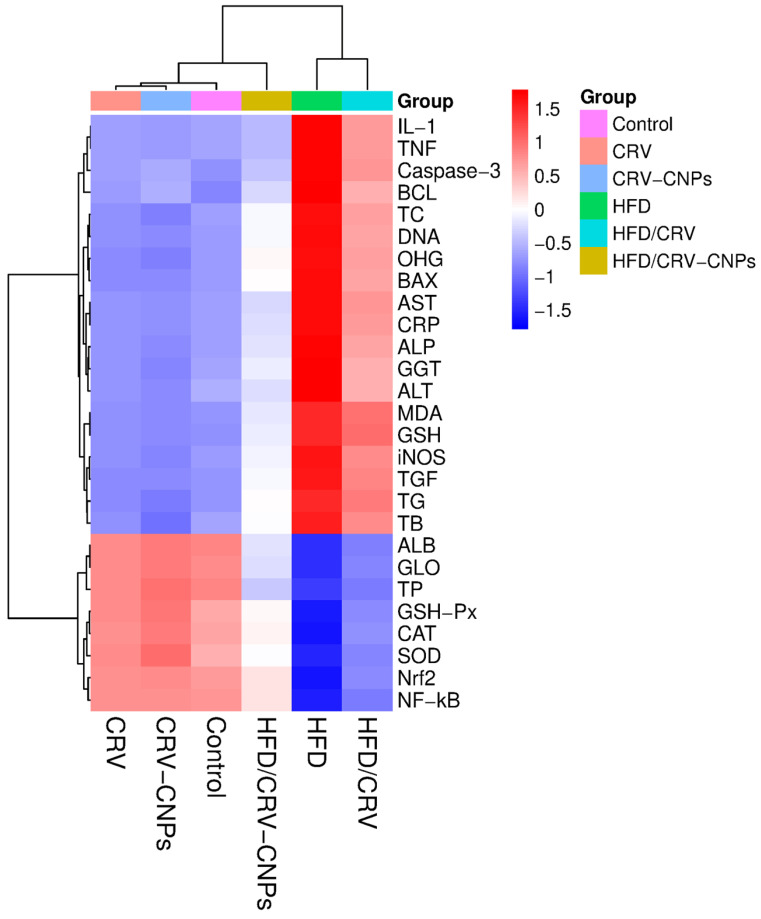
Hierarchical clustering heatmap of biochemical and molecular markers across experimental groups. Blue indicates lower levels; red indicates higher levels relative to the group mean. Key panels highlight proinflammatory cytokines (IL-1, TNF), apoptosis markers (Caspase-3, BAX, BCL), liver function enzymes (AST, ALT, ALP, GGT), oxidative stress markers (MDA, GSH), and regulatory proteins (Nrf2, NF-kB). CRV-CHNPs intervention notably reversed HFD-induced elevations in inflammatory and apoptotic markers, while restoring antioxidant defenses, as evidenced by marked color shifts in the corresponding clusters. These critical differences offer mechanistic insights into the therapeutic impact of CRV-CHNPs in NAFLD.

A hierarchical clustering heatmap (Figure 15) was generated to visualize the relationships among biochemical and molecular variables across all experimental groups. The control, CRV, and CRV-CNPs groups formed a closely related cluster, reflecting similar profiles characterized by lower expression of proinflammatory, apoptotic, and oxidative stress markers (IL-1β, TNF-α, Caspase-3, MDA, iNOS) and higher levels of antioxidant indicators (GSH, SOD, CAT, and Nrf2). In contrast, the HFD group formed a distinct, isolated cluster with the highest concentrations of inflammatory mediators, oxidative stress markers, and hepatic injury enzymes (ALT, AST, ALP, GGT), concurrent with diminished antioxidant defenses. The HFD + CRV and HFD + CRV-CNPs groups occupied intermediate positions between the HFD and control clusters, indicating partial restoration of biochemical and molecular homeostasis. Notably, the HFD + CRV-CNPs group showed the greatest alignment with the control-related cluster, underscoring the superior modulatory efficacy of the nanoformulation in restoring the hepatic redox-inflammatory balance.

## 4. Discussion

The present study provides mechanistic and therapeutic evidence for the hepatoprotective efficacy of carvacrol-loaded chitosan nanoparticles (CRV-CNPs) against high-fat diet (HFD)-induced liver injury in rats. Unlike previous reports that focused primarily on free carvacrol or other phytocompounds, our data demonstrate that nanoencapsulation markedly enhances carvacrol’s functional performance in mitigating hepatic steatosis, oxidative stress, inflammation, apoptosis, and genotoxicity.

### 4.1. Integration and Critical Comparison to Previous Work

Our molecular docking results elucidated specific interactions between carvacrol and antioxidant (Nrf2, SOD, CAT, GSH-Px), inflammatory (NF-κB, TNF-α, IL-1β, iNOS, TGF-β), and apoptotic (Bax, Bcl-2, caspase-3) pathways, providing a mechanistic rationale corroborated by comparable in silico studies for related monoterpenes and polyphenols [38,39]. However, prior work did not validate these computational predictions via direct in vivo assessment. Here, the alignment between docking and biological outcomes strengthens the translational value of our approach [40].

Long-term HFD feeding recapitulated key metabolic derangements seen in rodent NAFLD models, including weight gain, hyperlipidemia, and hepatic steatosis, consistent with previous studies [41,42]. The reduction in liver index and the restoration of ALT/AST observed following CRV-CNP administration closely match findings with silymarin, naringin, and curcumin-based nanoparticles in similar models, highlighting a common benefit of nanoencapsulation (i.e., enhanced hepatic protection) [43,44].

### 4.2. Lipid Metabolism and Antioxidant Restoration

CRV-CNPs normalized hepatic and serum lipid parameters and reduced histological steatosis. Previous work showed carvacrol could reduce lipid accumulation via AMPK-mediated β-oxidation and downregulation of SREBP-1c; our findings substantiate and extend these results by demonstrating that the nanocarrier formulation amplifies this effect and improves lipid export and antioxidant enzyme activity [12,14]. Comparable results were achieved with nanocarrier-formulated nutraceuticals, which significantly reduced lipid deposition and fibrosis in experimental NAFLD [45,46,47]. Thus, the metabolic normalization achieved by CRV-CNPs supports their translational potential as nano-nutraceuticals for NAFLD management [46].

Oxidative stress represents a central element in NAFLD pathogenesis, as hepatocyte fat overload increases mitochondrial ROS production, depleting endogenous antioxidant capacity [48]. Our data revealed elevated hepatic MDA levels and suppressed Nrf2 activation, SOD, CAT, and GSH-Px activities in HFD-fed rats, biochemical hallmarks of oxidative imbalance [49,50]. CRV-CNPs restored these parameters near baseline control values, confirming reactivation of the Nrf2-antioxidant axis, a key defense pathway also stimulated by other polyphenolic nanoparticles [51,52,53]. Recent nanobiomaterial studies are consistent with our findings, leading to reduced hepatic oxidative burden in NAFLD models [54].

### 4.3. Inflammation and Apoptosis

**Figure 16:**
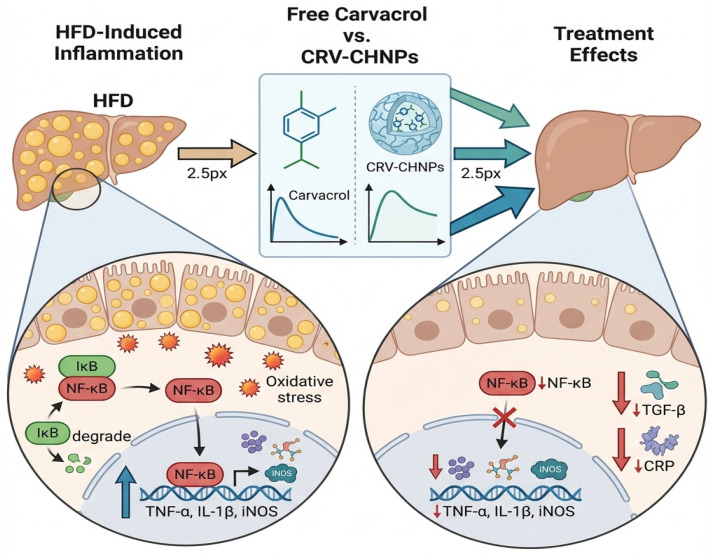
Molecular responses of inflammatory biomarkers to Carvacrol and CRV-CHNPs treatment in HFD-induced NAFLD Rat Model. Adapted from GRABSTRACT-Science, visualized (https://grabstract.io/) (accessed 20 November 2025).

Hepatic inflammation driven by NF-κB activation is a major contributor to NAFLD progression [55]. Elevated levels of NF-κB, TNF-α, IL-1β, and iNOS observed in our HFD model confirmed amplification of this pathway. CRV-CNPs markedly suppressed these proinflammatory mediators, outperforming free CRV (Figure 16). This effect likely arises from improved cellular internalization and sustained CRV release from the chitosan matrix. Similar declines in inflammatory signaling have been documented in studies where nanoparticle-encapsulated antioxidants, including curcumin-loaded PLGA and cerium oxide (CeO_2_) nanostructures, reduced hepatic cytokine release [56,57]. Furthermore, CRV-CNP administration reduced systemic CRP and TGF-β1 levels, reflecting attenuation of profibrotic processes by inhibiting hepatic stellate cell activation. This observation aligns with findings that nanocarrier-based antifibrotic systems mitigate collagen deposition by targeting ROS-TGF-β signaling [58,59,60,61].

NAFLD is also characterized by apoptosis and genotoxic stress resulting from lipotoxic metabolite accumulation and mitochondrial dysfunction. In this study, HFD significantly upregulated *Bax* and *Caspase-3*, while suppressing *Bcl-2*, leading to hepatocyte apoptosis. CRV-CNPs reversed this imbalance, restoring antiapoptotic signaling and reducing pro-apoptotic expression. These results concur with earlier studies where CRV modulated the Bcl-2/Bax ratio to prevent oxidative apoptosis [10,62]. In parallel, CRV-CNPs substantially decreased DNA fragmentation and 8-OHdG levels, confirming strong genomic protection. 8-OHdG is recognized as a reliable biomarker for oxidative DNA damage and NAFLD-associated carcinogenic risk [63,64]. Comparable reductions in 8-OHdG were reported with exosome-like, polyphenol-rich nanoparticles that enhanced Nrf2-mediated detoxifying enzyme expression and reduced hepatic vacuolation [65,66].

### 4.4. Histopathological and Ultrastructural Preservation

Histopathological and ultrastructural evaluations further supported these molecular findings. The HFD group displayed classical patterns of macrovesicular steatosis, lobular inflammation, and mitochondrial degeneration, consistent with previous reports linking lipid accumulation to mitochondrial cristae disruption and rER dilation [67,68]. Conversely, CRV-CNP administration largely preserved hepatocellular ultrastructure, maintaining intact mitochondria, organized rER, and normal nuclear morphology, confirming robust cytoprotective efficacy.

### 4.5. Multivariate Comparisons

The multivariate analysis, integrating biochemical, molecular, and histological parameters, underscored this effect, showing that the CRV-CNP treatment group clustered closest to the control, indicating near normalization of hepatic function and redox-inflammatory balance. This pattern mirrors previous multiscale omics-based clustering studies, demonstrating the restoration of transcriptional and metabolic signatures with targeted nanoformulations in NAFLD [69,70].

### 4.6. Innovative Mechanistic Insight

Collectively, the current data demonstrate that CRV-CNPs exert a comprehensive hepatoprotective effect through multi-mechanistic modulation of redox, inflammatory, apoptotic, and fibrotic pathways. By overcoming the solubility and stability limitations of free carvacrol, the chitosan nanoparticle matrix enables sustained therapeutic release, enhanced hepatocellular absorption, and improved pharmacodynamic efficacy.

The use of nanoparticles as carriers for carvacrol aims to enhance its hepatic bioavailability by protecting it from premature degradation in the gastrointestinal tract and facilitating its absorption. Upon oral administration, nanoparticles can enhance intestinal uptake via transcellular transport, paracellular diffusion, and endocytosis by enterocytes or M cells in Peyer’s patches. Once absorbed, these nanoparticles are often transported via the portal vein directly to the liver, thereby increasing local hepatic concentrations of the encapsulated compound. Recent studies indicate that the physicochemical properties of nanoparticles, including size, surface charge, and hydrophobicity, critically influence their interaction with the intestinal epithelium and subsequent systemic distribution [71,72]. Thus, the oral delivery of carvacrol-loaded nanoparticles represents a strategic approach to overcome the compound’s low intrinsic bioavailability, ensuring a more targeted and sustained release at the hepatic level. Integrating these findings with prior reports on nanocarrier-based antioxidants, such as silymarin-, curcumin-, and naringenin-loaded nanoparticles, suggests that polymeric nanoformulations represent a viable strategy for next-generation NAFLD therapeutics [40,73,74].

### 4.7. Study Limitations and Future Perspectives

Despite providing valuable mechanistic insights, this study has certain limitations that should be acknowledged. First, using a single animal model and dosage regimen may not fully recapitulate the metabolic heterogeneity observed in human NAFLD. Additional models incorporating genetic susceptibility or comorbidities, such as insulin resistance or obesity, would strengthen translational relevance. Second, although CRV-CNPs demonstrated favorable biochemical and histological outcomes, long-term pharmacokinetic, biodistribution, and toxicity profiles were not evaluated; these are crucial to establishing clinical safety, as prolonged nanoparticle accumulation could pose hepatic or immunological risks. While chitosan-based nanoparticles are generally considered biocompatible and biodegradable according to previous studies, comprehensive in vivo safety evaluations, including potential immunogenicity and long-term tissue accumulation, are essential before advancing to human use.

Third, the study did not include in-depth molecular or omics-based analyses to delineate global transcriptional or metabolic alterations downstream of Nrf2 or NF-κB signaling. As emphasized in recent nanotherapeutic reviews, multi-omics are essential for validating molecular targets and clarifying nanoparticle–cell interactions in liver tissue. Furthermore, it should be noted that all experimental methods carry inherent uncertainties, and the observed data may be influenced by technical variability or biological heterogeneity; therefore, interpretations should be made cautiously.

Future studies should, therefore, explore dose–response optimization, long-term safety under chronic exposure conditions, and combinatory approaches involving CRV-CNPs with other hepatoprotective nutraceuticals or metabolic modulators. Ultimately, translating these findings into clinical intervention will necessitate GMP-scale production, pharmacokinetic modeling, and targeted delivery validation to ensure reproducibility and patient safety in human NAFLD [75].

## 5. Conclusions

This study demonstrates that carvacrol-loaded chitosan nanoparticles (CRV-CNPs) provide potent hepatoprotection against HFD-induced nonalcoholic fatty liver disease by targeting multiple pathogenic mechanisms. CRV-CNPs effectively alleviated hepatic lipid accumulation, oxidative stress, inflammation, apoptosis, and DNA damage while maintaining normal liver architecture. The enhanced efficacy of CRV-CNPs is attributed to improved solubility, stability, and bioavailability, enabling sustained hepatic delivery. Mechanistically, CRV-CNPs upregulated hepatic Nrf2 and antioxidant enzymes, inhibited NF-κB signaling and downstream inflammatory cytokines (TNF-α, IL-1β, iNOS), attenuated pro-apoptotic gene expression (Bax, Caspase-3), and prevented oxidative DNA damage, thereby restoring biochemical balance and preserving cellular integrity more efficiently than free CRV. Together, these findings highlight CRV-CNPs as a promising nanotherapeutic platform for NAFLD management, integrating antioxidant, anti-inflammatory, and antiapoptotic properties within a safe, biocompatible design. Further studies should evaluate pharmacokinetics, long-term safety, and clinical translation.

## Figures and Tables

**Table 1:** Primer sequences for quantitative RT-PCR analysis of apoptosis-related genes in Wistar rat liver.

Gene	* NCBI Gene ID_Rattus Norvegicus	Forward (5′→3′)	Reverse (5′→3′)
*Bax*	24887	CGGCGAATTGGAGATGAACTGG	CTAGCAAAGTAGAAGAGGGCAACC
*Bcl-2*	24224	TGTGGATGACTGACTACCTGAACC	CAGCCAGGAGAAATCAAACAGAGG
*Caspase-3*	25402	GTGGAACTGACGATGATATGGC	CGCAAAGTGACTGGATGAACC
*β-Actin*	81822	AAGATCCTGACCGAGCGTGG	CAGCACTGTGTTGGCATAGAGG

* NCBI: National Center for Biotechnology Information available at (https://www.ncbi.nlm.nih.gov/) (last accessed 28 October 2025).

**Table 2:** Scoring system for semi-quantitative liver lesion assessment.

Score	Composite Multi-Criteria Liver Assessment Score
0 (none)	No pathological changes
1 (mild)	The liver demonstrated sporadic to mild hepatocellular degeneration and necrosis, accompanied by little to no inflammatory cell infiltration and occasional vascular congestion.
2 (moderate)	The liver showed marked inflammatory cell infiltration, moderate vacuolar hepatocyte degeneration, mild vascular congestion, and multifocal necrotic lesions.
3 (severe)	The liver demonstrated extensive inflammatory infiltration and necrosis, along with severe degeneration of hepatocytes and moderate to severe vascular congestion.

**Table 3:** Molecular docking interactions of Carvacrol with key antioxidant, inflammatory, apoptotic, and fibrogenic targets involved in hepatic protection.

Target	Affinity (kcal/mol)	Hydrogen Bonds	Hydrophobic Contacts	Active Residues
Nrf2	−5.7	4	2	Val606(2), Gly367(2), Ala366, Val418
CAT	−5	1	3	Arg363, His364(2), Pro368
SOD	−5.4	2	4	Val7(2), Cys6, Asn53(2), Val148
GSH-Px	−6	0	6	Glu88(2), Phe103(2), Arg106(2)
NF-қB	−6.3	0	8	Trp464(3), Arg432(2), Lys430(2), Glu413
TNF-α	−5.6	0	7	Tyr119, Tyr59(3), Leu57(3)
Il-1β	−4.6	0	7	Phe150(2), Met148, Leu110(2), Lys103, Thr147
Caspase-3	−4.1	0	5	Lys137, Tyr195, Tyr197, Val266(2)
iNOS	−4.8	0	4	Tyr270, Ile291, Pro273, Tyr299
TGF-β	−4.1	1	5	Asn103, Ala41, Ile22, Leu28, Trp30(2)

The table summarizes binding affinity (kcal/mol), the number of hydrogen bonds and hydrophobic interactions, and the major active-site residues contributing to ligand stabilization for each protein. Nrf2: Nuclear factor erythroid 2–related factor 2; CAT: catalase; SOD: superoxide dismutase; GSH-Px: glutathione peroxidase; NF-κB: Nuclear Factor kappa-light-chain-enhancer of activated B cells; TNF-α: Tumor Necrosis Factor-alpha; IL-1β: Interleukin-1β; iNOs: inducible nitric oxide synthase; TGF-β: transforming growth factor-beta.

**Table 4:** Effects of carvacrol (CRV) and CRV-loaded chitosan nanoparticles (CRV-CNPs) on hepatic function and lipid metabolism indices in rats fed a high-fat diet.

Items	Control	CRV	CRV-CNPs	HFD	HFD/CRV	HFD/CRV-CNPs
TP (g/dL)	5.98 ± 0.38 **^#^**	6.01 ± 0.42 **^#^**	6.13 ± 0.41 **^#^**	3.22 ± 0.33	3.95 ± 0.29	4.72 ± 0.49 **^#,¥^**
Alb (g/dL)	3.17 ± 0.15 **^#^**	3.12 ± 0.11 **^#^**	3.19 ± 0.28 **^#^**	1.72 ± 0.09	2.01 ± 0.14	2.53 ± 0.13 *^,#,¥^
Glo (g/dL)	2.81 ± 0.16 **^#^**	2.89 ± 0.18 **^#^**	2.94 ± 0.20 **^#^**	1.53 ± 0.12	1.94 ± 0.09	2.19 ± 0.07 **^#,¥^**
ALT (U/L)	34.21 ± 3.55 **^#^**	31.20 ± 2.31 **^#^**	30.03 ± 3.59 **^#^**	88.21 ± 5.77	65.59 ± 4.16 **^#^**	41.07 ± 3.38 *^#^
AST (U/L)	71.13 ± 4.47 **^#^**	70.13 ± 5.18 **^#^**	68.52 ± 3.62 **^#^**	141.91 ± 8.11	112.70 ± 7.16 **^#^**	84.54 ± 4.25 *^#^
ALP (U/L)	153.21 ± 6.93 **^#^**	149.84 ± 5.08 **^#^**	147.61 ± 6.17 **^#^**	220.36 ± 10.37	184.16 ± 7.45 **^#^**	166.27 ± 5.20 **^#^**
GGT (U/L)	11.21 ± 1.12 **^#^**	10.41 ± 2.14 **^#^**	9.27 ± 1.40 **^#^**	22.17 ± 3.02	18.41 ± 1.76 **^#^**	14.33 ± 2.43 **^#^**
TB (g/dL)	0.41 ± 0.06 **^#^**	0.39 ± 0.08 **^#^**	0.38 ± 0.04 **^#^**	1.32 ± 0.12	0.87 ± 0.10 **^#^**	0.62 ± 0.09 **^#^**
TG (mg/dL)	57.12 ± 4.11 **^#^**	55.39 ± 3.23 **^#^**	52.28 ± 5.12 **^#^**	123.31 ± 6.18	105.58 ± 4.57 **^#^**	79.77 ± 3.62 *^,#,¥^
TC (mg/dL)	78.26 ± 5.42 **^#^**	74.12 ± 6.18 **^#^**	72.58 ± 4.77 **^#^**	131.22 ± 8.26	98.16 ± 4.11 **^#^**	86.27 ± 4.09 **^#^**

Data are expressed as mean ± SE. Abbreviations: “TG, triglycerides; TC, total cholesterol; ALT, alanine aminotransferase; AST, aspartate aminotransferase; ALP, alkaline phosphatase; GGT, gamma-glutamyltransferase; TB, Total bilirubin; TP, total protein; Alb, albumin; Glo, globulin.” CRV: carvacrol (100 mg/kg body weight); CRV-CNPs: carvacrol-loaded chitosan nanoparticles (100 mg/kg body weight); HFD: high-fat diet; HFD/CRV: carvacrol (100 mg/kg body weight) + high-fat diet; HFD/CRV-CNPs: carvacrol-loaded chitosan nanoparticles (100 mg/kg body weight) + high-fat diet. ^#^ indicates significant difference compared to the HFD group; * indicates significant difference compared to the HFD/CRV-CNPs group; ^¥^ indicates significant difference compared to the Control group.

## Data Availability

The original contributions presented in this study are included in the article. Further inquiries can be directed to the corresponding author.

## Abbreviations

The following abbreviations are used in this manuscript:

AASLD	American Association for the Study of Liver Diseases
ALB	Albumin
ALP	Alkaline phosphatase
ALT	Alanine aminotransferase
AMPK	AMP-activated protein kinase
AST	Aspartate aminotransferase
Bax	Bcl-2-associated X protein
Bcl-2	B-cell lymphoma 2
CAT	Catalase
Caspase-3	Cysteine–aspartic acid protease-3
CH	Chitosan
CRP	C-reactive protein
CRV	Carvacrol
CRV-CNPs	Carvacrol-loaded chitosan nanoparticles
DLS	Dynamic light scattering
DNA	Deoxyribonucleic acid
ELISA	Enzyme-linked immunosorbent assay
EMA	European Medicines Agency
FAO	Food and Agriculture Organization
GGT	Gamma-glutamyl transferase
GMP	Good Manufacturing Practice
GSH	Reduced glutathione
GSH-Px	Glutathione peroxidase
HDL	High-density lipoprotein
HFD	High-fat diet
HSCs	Hepatic stellate cells
H&E	Hematoxylin and eosin
IL-1β	Interleukin-1 beta
iNOS	Inducible nitric oxide synthase
LD	Lipid droplets
LC	Loading content
LE	Loading efficiency
LDL	Low-density lipoprotein
MDA	Malondialdehyde
NAFLD	Nonalcoholic fatty liver disease
NASH	Non-alcoholic steatohepatitis
NF-κB	Nuclear factor kappa B
NIH	National Institutes of Health
Nrf2	Nuclear factor erythroid 2–related factor 2
PDI	Polydispersity index
qRT-PCR	Quantitative real-time polymerase chain reaction
RNA	Ribonucleic acid
ROS	Reactive oxygen species
SOD	Superoxide dismutase
SRplot	Science and Research Plotting Tool
TB	Total bilirubin
TC	Total cholesterol
TEM	Transmission electron microscopy
TG	Triglycerides
TGF-β	Transforming growth factor-beta
TNF-α	Tumor necrosis factor-alpha

## References

[1] Han S.K., Baik S.K., Kim M.Y. (2023). Non-alcoholic fatty liver disease: Definition and subtypes. Clin. Mol. Hepatol..

[2] Kim Y., Chang Y., Cho Y.K., Ahn J., Shin H., Ryu S. (2019). Metabolically healthy versus unhealthy obesity and risk of fibrosis progression in non-alcoholic fatty liver disease. Liver Int. Off. J. Int. Assoc. Study Liver.

[3] Bence K.K., Birnbaum M.J. (2021). Metabolic drivers of non-alcoholic fatty liver disease. Mol. Metab..

[4] Pierantonelli I., Svegliati-Baroni G. (2019). Nonalcoholic Fatty Liver Disease: Basic Pathogenetic Mechanisms in the Progression From NAFLD to NASH. Transplantation.

[5] Salvoza N., Giraudi P.J., Tiribelli C., Rosso N. (2022). Natural Compounds for Counteracting Nonalcoholic Fatty Liver Disease (NAFLD): Advantages and Limitations of the Suggested Candidates. Int. J. Mol. Sci..

[6] Hallsworth K., McPherson S., Anstee Q.M., Flynn D., Haigh L., Avery L. (2021). Digital Intervention With Lifestyle Coach Support to Target Dietary and Physical Activity Behaviors of Adults With Nonalcoholic Fatty Liver Disease: Systematic Development Process of VITALISE Using Intervention Mapping. J. Med. Internet Res..

[7] Nendouvhada L.P., Sibuyi N.R.S., Fadaka A.O., Meyer S., Madiehe A.M., Meyer M., Gabuza K.B. (2024). Phytonanotherapy for the Treatment of Metabolic Dysfunction-Associated Steatotic Liver Disease. Int. J. Mol. Sci..

[8] Wong V.W., Singal A.K. (2019). Emerging medical therapies for non-alcoholic fatty liver disease and for alcoholic hepatitis. Transl. Gastroenterol. Hepatol..

[9] Wal P., Yadav S., Jha S.K., Singh A., Bhargavi B., Shivaram R., Imran M., Aziz N. (2025). Role of natural compounds in non-alcoholic fatty liver diseases (NAFLD): A mechanistic approach. Egypt. Liver J..

[10] Sadeghzadeh S., Hejazian S.H., Jamhiri M., Hafizibarjin Z., Sadeghzadeh S., Safari F. (2018). The effect of carvacrol on transcription levels of Bcl-2 family proteins in hypertrophied heart of rats. Physiol. Pharmacol..

[11] Mohseni R., Karimi J., Tavilani H., Khodadadi I., Hashemnia M. (2020). Carvacrol Downregulates Lysyl Oxidase Expression and Ameliorates Oxidative Stress in the Liver of Rats with Carbon Tetrachloride-Induced Liver Fibrosis. Indian. J. Clin. Biochem..

[12] Khazdair M.R., Moshtagh M., Anaeigoudari A., Jafari S., Kazemi T. (2024). Protective effects of carvacrol on lipid profiles, oxidative stress, hypertension, and cardiac dysfunction–A comprehensive review. Food Sci. Nutr..

[13] Mączka W., Twardawska M., Grabarczyk M., Wińska K. (2023). Carvacrol-A Natural Phenolic Compound with Antimicrobial Properties. Antibiotics.

[14] Cho S., Choi Y., Park S., Park T. (2012). Carvacrol prevents diet-induced obesity by modulating gene expressions involved in adipogenesis and inflammation in mice fed with high-fat diet. J. Nutr. Biochem..

[15] Vitali A., Stringaro A., Colone M., Muntiu A., Angiolella L. (2021). Antifungal Carvacrol Loaded Chitosan Nanoparticles. Antibiotics.

[16] Llana-Ruiz-Cabello M., Gutiérrez-Praena D., Pichardo S., Moreno F.J., Bermúdez J.M., Aucejo S., Cameán A.M. (2014). Cytotoxicity and morphological effects induced by carvacrol and thymol on the human cell line Caco-2. Food Chem. Toxicol..

[17] Mauriello E., Ferrari G., Donsì F. (2021). Effect of formulation on properties, stability, carvacrol release and antimicrobial activity of carvacrol emulsions. Colloids Surf. B Biointerfaces.

[18] Tripathy A., Pahal S., Mudakavi R.J., Raichur A.M., Varma M.M., Sen P. (2018). Impact of Bioinspired Nanotopography on the Antibacterial and Antibiofilm Efficacy of Chitosan. Biomacromolecules.

[19] Garg U., Chauhan S., Nagaich U., Jain N. (2019). Current Advances in Chitosan Nanoparticles Based Drug Delivery and Targeting. Adv. Pharm. Bull..

[20] Barbosa M., Vale N., Costa F.M.T.A., Martins M.C.L., Gomes P. (2017). Tethering antimicrobial peptides onto chitosan: Optimization of azide-alkyne “click” reaction conditions. Carbohydr. Polym..

[21] Layek B., Lipp L., Singh J. (2015). Cell Penetrating Peptide Conjugated Chitosan for Enhanced Delivery of Nucleic Acid. Int. J. Mol. Sci..

[22] Ewii U.E., Attama A.A., Olorunsola E.O., Onugwu A.L., Nwakpa F.U., Anyiam C., Chijioke C., Ogbulie T. (2025). Nanoparticles for drug delivery: Insight into in vitro and in vivo drug release from nanomedicines. Nano TransMed.

[23] Mortazavi A., Mohammad Pour Kargar H., Beheshti F., Anaeigoudari A., Vaezi G., Hosseini M. (2023). The effects of carvacrol on oxidative stress, inflammation, and liver function indicators in a systemic inflammation model induced by lipopolysaccharide in rats. Int. J. Vitam. Nutr. Res..

[24] Badr A.M., El-Orabi N.F., Mahran Y.F., Badr A.M., Bayoumy N.M., Hagar H., Elmongy E.I., Atawia R.T. (2023). In vivo and In silico evidence of the protective properties of carvacrol against experimentally-induced gastric ulcer: Implication of antioxidant, anti-inflammatory, and antiapoptotic mechanisms. Chem. Biol. Interact..

[25] Hosseini M., Arab Z., Beheshti F., Anaeigoudari A., Shakeri F., Rajabian A. (2023). Zataria multiflora and its constituent, carvacrol, counteract sepsis-induced aortic and cardiac toxicity in rat: Involvement of nitric oxide and oxidative stress. Anim. Models Exp. Med..

[26] Chang K.-J., Lin J.-A., Chen S.-Y., Weng M.-H., Yen G.-C. (2019). Silymarin protects against high fat diet-evoked metabolic injury by induction of glucagon-like peptide 1 and sirtuin 1. J. Funct. Foods.

[27] Cui X.S., Li H.Z., Li L., Xie C.Z., Gao J.M., Chen Y.Y., Zhang H.Y., Hao W., Fu J.H., Guo H. (2025). Rodent model of metabolic dysfunction-associated fatty liver disease: A systematic review. J. Gastroenterol. Hepatol..

[28] Zhang X., Hartmann P. (2023). How to calculate sample size in animal and human studies. Front. Med..

[29] Festing M.F.W., Altman D.G. (2002). Guidelines for the Design and Statistical Analysis of Experiments Using Laboratory Animals. ILAR J..

[30] Xu Z.J., Fan J.G., Ding X.D., Qiao L., Wang G.L. (2010). Characterization of high-fat, diet-induced, non-alcoholic steatohepatitis with fibrosis in rats. Dig. Dis. Sci..

[31] Krohn R.I. (2011). The colorimetric detection and quantitation of total protein. Curr. Protoc. Cell Biol..

[32] Goodla L., Manubolu M., Pathakoti K., Jayakumar T., Sheu J.-R., Fraker M., Tchounwou P.B., Poondamalli P.R. (2019). Protective Effects of Ammannia baccifera Against CCl4-Induced Oxidative Stress in Rats. Int. J. Environ. Res. Public Health.

[33] Rosenthal P., Blanckaert N., Kabra P.M., Thaler M.M. (1981). Liquid-chromatographic determination of bilirubin and its conjugates in rat serum and human amniotic fluid. Clin. Chem..

[34] Hadi W.H., Khudair T.T., Al-Fartosi K.G. (2023). Level of lipid profile and liver enzyme of diabetic male rats induced by streptozotocin treated with forxiga. 3c Empresa Investig. Y Pensam. Crítico.

[35] Livak K.J., Schmittgen T.D. (2001). Analysis of relative gene expression data using real-time quantitative PCR and the 2^−ΔΔCT^ Method. Methods.

[36] Graham L., Orenstein J.M. (2007). Processing tissue and cells for transmission electron microscopy in diagnostic pathology and research. Nat. Protoc..

[37] Wisse E., Braet F., Duimel H., Vreuls C., Koek G., Olde Damink S.W., van den Broek M.A., De Geest B., Dejong C.H., Tateno C. (2010). Fixation methods for electron microscopy of human and other liver. World J. Gastroenterol..

[38] Shinde P., Agraval H., Srivastav A.K., Yadav U.C.S., Kumar U. (2020). Physico-chemical characterization of carvacrol loaded zein nanoparticles for enhanced anticancer activity and investigation of molecular interactions between them by molecular docking. Int. J. Pharm..

[39] Olaokun O. (2025). Computational identification of polyphenols from medicinal plant extracts with biological activity against oxidative stress-related diseases: An in silico anti-inflammatory study. S. Afr. J. Bot..

[40] Moghtadaie A., Mahboobi H., Fatemizadeh S., Kamal M.A. (2023). Emerging role of nanotechnology in treatment of non-alcoholic fatty liver disease (NAFLD). EXCLI J..

[41] Lian C.-Y., Zhai Z.-Z., Li Z.-F., Wang L. (2020). High fat diet-triggered non-alcoholic fatty liver disease: A review of proposed mechanisms. Chem. Biol. Interact..

[42] Estévez-Vázquez O., Benedé-Ubieto R., Guo F., Gómez-Santos B., Aspichueta P., Reissing J., Bruns T., Sanz-García C., Sydor S., Bechmann L.P. (2021). Fat: Quality, or Quantity? What Matters Most for the Progression of Metabolic Associated Fatty Liver Disease (MAFLD). Biomedicines.

[43] Bakır M., Geyikoglu F., Colak S., Turkez H., Bakır T.O., Hosseinigouzdagani M. (2016). The carvacrol ameliorates acute pancreatitis-induced liver injury via antioxidant response. Cytotechnology.

[44] Mohebbati R., Paseban M., Soukhtanloo M., Jalili-Nik M., Shafei M.N., Yazdi A.J., Rad A.K. (2018). Effects of standardized Zataria multiflora extract and its major ingredient, Carvacrol, on Adriamycin-induced hepatotoxicity in rat. Biomed. J..

[45] Abou Assi R., Abdulbaqi I.M., Siok Yee C. (2021). The Evaluation of Drug Delivery Nanocarrier Development and Pharmacological Briefing for Metabolic-Associated Fatty Liver Disease (MAFLD): An Update. Pharmaceuticals.

[46] Moosavian S.A., Sathyapalan T., Jamialahmadi T., Sahebkar A. (2021). The Emerging Role of Nanomedicine in the Management of Nonalcoholic Fatty Liver Disease: A State-of-the-Art Review. Bioinorg. Chem. Appl..

[47] Li S., Duan F., Li S., Lu B. (2024). Administration of silymarin in NAFLD/NASH: A systematic review and meta-analysis. Ann. Hepatol..

[48] Arroyave-Ospina J.C., Wu Z., Geng Y., Moshage H. (2021). Role of Oxidative Stress in the Pathogenesis of Non-Alcoholic Fatty Liver Disease: Implications for Prevention and Therapy. Antioxidants.

[49] Li S., Tan H.-Y., Wang N., Zhang Z.-J., Lao L., Wong C.-W., Feng Y. (2015). The role of oxidative stress and antioxidants in liver diseases. Int. J. Mol. Sci..

[50] Li N., Hao L., Li S., Deng J., Yu F., Zhang J., Nie A., Hu X. (2024). The NRF-2/HO-1 Signaling Pathway: A Promising Therapeutic Target for Metabolic Dysfunction-Associated Steatotic Liver Disease. J. Inflamm. Res..

[51] Muriel P., Ramos-Tovar E., Montes-Páez G., Buendía-Montaño L.D. (2017). Experimental Models of Liver Damage Mediated by Oxidative Stress.

[52] Lebda M.A., Sadek K.M., Abouzed T.K., Tohamy H.G., El-Sayed Y.S. (2018). Melatonin mitigates thioacetamide-induced hepatic fibrosis via antioxidant activity and modulation of proinflammatory cytokines and fibrogenic genes. Life Sci..

[53] Ngo V., Duennwald M.L. (2022). Nrf2 and Oxidative Stress: A General Overview of Mechanisms and Implications in Human Disease. Antioxidants.

[54] Padmanaban S., Pully D., Samrot A.V., Gosu V., Sadasivam N., Park I.K., Radhakrishnan K., Kim D.K. (2023). Rising Influence of Nanotechnology in Addressing Oxidative Stress-Related Liver Disorders. Antioxidants.

[55] Kumar D., Dwivedi D.K., Lahkar M., Jangra A. (2019). Hepatoprotective potential of 7,8-Dihydroxyflavone against alcohol and high-fat diet induced liver toxicity via attenuation of oxido-nitrosative stress and NF-κB activation. Pharmacol. Rep..

[56] Karatayli E., Sadiq S.C., Schattenberg J.M., Grabbe S., Biersack B., Kaps L. (2025). Curcumin and Its Derivatives in Hepatology: Therapeutic Potential and Advances in Nanoparticle Formulations. Cancers.

[57] Casals G., Perramón M., Casals E., Portolés I., Fernández-Varo G., Morales-Ruiz M., Puntes V., Jiménez W. (2021). Cerium Oxide Nanoparticles: A New Therapeutic Tool in Liver Diseases. Antioxidants.

[58] Singh S., Sharma N., Shukla S., Behl T., Gupta S., Anwer M.K., Vargas-De-La-Cruz C., Bungau S.G., Brisc C. (2023). Understanding the Potential Role of Nanotechnology in Liver Fibrosis: A Paradigm in Therapeutics. Molecules.

[59] Chen Y.N., Li M.Q., Zhang H.J., Xu N.N., Xu Y.Q., Liu W.X., Chen T.T., Li N., Wu G.Y., Zhao J.M. (2025). Nanoparticle-based drug delivery systems: A promising approach for the treatment of liver fibrosis. Int. J. Pharm X.

[60] Du X., Niu R., Liu X., Wu F., Yang X., Ma X., Zhang J., Zhou H., Shao L., Wang S. (2025). Nanomedicines in the Treatment of Liver Fibrosis: A Review. Int. J. Nanomed..

[61] Mostafa S., Shetab Boushehri M.A., Ezzat A.A., Weiskirchen R., Lamprecht A., Mansour S., Tammam S.N. (2025). Targeted Delivery of Anti-TGF-β1-siRNA Using PDGFR-β Peptide-Modified Chitosan Nanoparticles for the Treatment of Liver Fibrosis. Mol. Pharm..

[62] Dariushnejad H., Roshanravan N., Wasman H.M., Cheraghi M., Pirzeh L., Ghorbanzadeh V. (2024). Silibinin, Synergistically Enhances Vinblastine-Mediated Apoptosis in Triple Negative Breast Cancer Cell Line: Involvement of Bcl2/Bax Caspase-3 Pathway. Int. J. Hematol. Oncol. Stem Cell Res..

[63] Dwivedi D.K., Jena G.B. (2020). NLRP3 inhibitor glibenclamide attenuates high-fat diet and streptozotocin-induced non-alcoholic fatty liver disease in rat: Studies on oxidative stress, inflammation, DNA damage and insulin signalling pathway. Naunyn-Schmiedeberg’s Arch. Pharmacol..

[64] Omari Shekaftik S., Nasirzadeh N. (2021). 8-Hydroxy-2’-deoxyguanosine (8-OHdG) as a biomarker of oxidative DNA damage induced by occupational exposure to nanomaterials: A systematic review. Nanotoxicology.

[65] Ma-On C., Sanpavat A., Whongsiri P., Suwannasin S., Hirankarn N., Tangkijvanich P., Boonla C. (2017). Oxidative stress indicated by elevated expression of Nrf2 and 8-OHdG promotes hepatocellular carcinoma progression. Med. Oncol..

[66] Subudhi P.D., Bihari C., Sarin S.K., Baweja S. (2022). Emerging role of edible exosomes-like nanoparticles (ELNs) as hepatoprotective agents. Nanotheranostics.

[67] Zheng P., Ma W., Gu Y., Wu H., Bian Z., Liu N., Yang D., Chen X. (2023). High-fat diet causes mitochondrial damage and downregulation of mitofusin-2 and optic atrophy-1 in multiple organs. J. Clin. Biochem. Nutr..

[68] Xue R., Wu Q., Guo L., Ye D., Cao Q., Zhang M., Xian Y., Chen M., Yan K., Zheng J. (2024). Pyridostigmine attenuated high-fat-diet induced liver injury by the reduction of mitochondrial damage and oxidative stress via α7nAChR and M3AChR. J. Biochem. Mol. Toxicol..

[69] Ægidius H.M., Veidal S.S., Feigh M., Hallenborg P., Puglia M., Pers T.H., Vrang N., Jelsing J., Kornum B.R., Blagoev B. (2020). Multi-omics characterization of a diet-induced obese model of non-alcoholic steatohepatitis. Sci. Rep..

[70] Ramos-Lopez O. (2022). Multi-Omics Nutritional Approaches Targeting Metabolic-Associated Fatty Liver Disease. Genes.

[71] Sabourian P., Yazdani G., Ashraf S.S., Frounchi M., Mashayekhan S., Kiani S., Kakkar A. (2020). Effect of Physico-Chemical Properties of Nanoparticles on Their Intracellular Uptake. Int. J. Mol. Sci..

[72] Zheng Y., Luo S., Xu M., He Q., Xie J., Wu J., Huang Y. (2024). Transepithelial transport of nanoparticles in oral drug delivery: From the perspective of surface and holistic property modulation. Acta Pharm. Sinica B.

[73] Hegazi O.E., Alalalmeh S.O., Alnuaimi G.R.H., Shahwan M., Jairoun A.A., Alorfi N.M., Majrashi S.A., Alkhanani M.F., Alkhattabi A., Alourfi M.M. (2023). NAFLD and nutraceuticals: A review of completed phase III and IV clinical trials. Front. Med..

[74] Li G., Dai Z., Guo J. (2025). Therapeutic Nanomaterials in NAFLD: Current Advances and Potential Applications in Patients with Concurrent HBV Infection. Int. J. Nanomed..

[75] Padmanaban S., Baek J.-W., Chamarthy S.S., Chandrasekaran S., Samrot A.V., Gosu V., Park I.-K., Radhakrishnan K., Kim D.-K. (2025). Nanoparticle-based therapeutic strategies for chronic liver diseases: Advances and insights. Liver Res..

